# Characteristics of a root hair-less line of *Arabidopsis thaliana* under physiological stresses

**DOI:** 10.1093/jxb/eru014

**Published:** 2014-02-05

**Authors:** Natsuki Tanaka, Mariko Kato, Rie Tomioka, Rie Kurata, Yoichiro Fukao, Takashi Aoyama, Masayoshi Maeshima

**Affiliations:** ^1^Graduate School of Bioagricultural Sciences, Nagoya University, Nagoya, 464–8601, Japan; ^2^Institute for Chemical Research, Kyoto University, Gokasho, Uji, Kyoto, 611-0011, Japan; ^3^Graduate School of Biological Sciences, Nara Institute of Science and Technology, Ikoma, 630-0192, Japan

**Keywords:** Mineral nutrients, phosphate, root anchoring, root hair, stress tolerance, water absorption.

## Abstract

The plasma membrane-associated Ca^2+^-binding protein-2 of *Arabidopsis thaliana* is involved in the growth of root hair tips. Several transgenic lines that overexpress the 23 residue N-terminal domain of this protein under the control of the root hair-specific *EXPANSIN A7* promoter lack root hairs completely. The role of root hairs under normal and stress conditions was examined in one of these root hair-less lines (NR23). Compared with the wild type, NR23 showed a 47% reduction in water absorption, decreased drought tolerance, and a lower ability to adapt to heat. Growth of NR23 was suppressed in media deficient in phosphorus, iron, calcium, zinc, copper, or potassium. Also, the content of an individual mineral in NR23 grown in normal medium, or in medium lacking a specific mineral, was relatively low. In wild-type plants, the primary and lateral roots produce numerous root hairs that become elongated under phosphate-deficient conditions; NR23 did not produce root hairs. Although several isoforms of the plasma membrane phosphate transporters including PHT1;1–PHT1;6 were markedly induced after growth in phosphate-deficient medium, the levels induced in NR23 were less than half those observed in the wild type. In phosphate-deficient medium, the amounts of acid phosphatase, malate, and citrate secreted from NR23 roots were 38, 9, and 16% of the levels secreted from wild-type roots. The present results suggest that root hairs play significant roles in the absorption of water and several minerals, secretion of acid phosphatase(s) and organic acids, and in penetration of the primary roots into gels.

## Summary

Root hairs of *Arabidopsis* play significant roles in the absorption of water and several minerals, secretion of acid phosphatases and organic acids, and anchoring of roots.

## Introduction

Root hairs, which are specialized epidermal cells with a large surface area exposed to soil, exhibit tip growth similar to pollen tubes. The unique properties of root hairs in cell differentiation and development have attracted considerable attention, particularly the molecular mechanisms associated with bulge formation (initial stage of root hair formation) and the tip growth of root hairs (Datta *et al.*, 2011; Grebe, 2012; Kwasniewski *et al.*, 2013). The polarity of the cell membrane during elongation is regulated by a robust mechanism involving highly polarized membrane traffic, cytoskeletal reorganization, and calcium signalling at the tip (Smith and Oppenheimer, 2005; Cole and Fowler, 2006; Campanoni and Blatt, 2007; Datta *et al.*, 2011).

Root hairs have been suggested to play an important role in the absorption of nutrients and water, and also in root anchoring at the elongation stage (for reviews, see Gilroy and Jones, 2000; Emons and Ketelaar, 2009; Libault *et al.*, 2010; Datta *et al.*, 2011). Consequently, elongating root hairs are highly active in cell growth and in the absorption of nutrients and water, which are transported to cells of the root endodermis and then to shoots. Molecular biological analyses of root hairs have identified several root hair-specific genes, such as *RHD6* (At1G66470; transcription factor), *RHD2* (At5G51060; signalling protein), and *EXPA7* (At1g12560; expansin) (Cho, 2013). At present, these transcription factors and signal transducers involved in root hair differentiation and development have been identified (Jones *et al.*, 2002; Ishida *et al.*, 2008; Datta *et al.*, 2011; Yi *et al.*, 2010). For membrane transporters, few root hair-specific genes or gene products, which are closely related to root hair functions, have been detected; the reason for this small number of reports is primarily due to the technical difficulties associated with the preparation of root hair cells, including non-elongating trichoblasts, bulge-forming cells, and mature root hair cells. Consequently, mutant and transgenic lines with different root hair phenotypes provide a means for studying the physical and physiological contributions of root hairs to the plant life cycle.

The physiological significance of root hairs has been examined by comparing the physiological phenotypes of wild type (WT) plants against mutant phenotypes having abnormal root hair emergence or elongation characteristics. Here mutants of *Arabidopsis thaliana* that have such root hair phenotypes are briefly introduced, although several studies have reported the physiological roles of root hairs in crops such as barley (Gahoonia *et al.*, 2001; Gahoonia and Nielsen, 2003; Brown *et al.*, 2012). A double mutant of the transcription factors CPC and TRY (*cpc try*) was reported to have abnormal trichomes and no root hairs (Schellmann *et al.*, 2002). In another transcription factor RHD6 (*rhd6*) mutant that has few root hairs, ethylene treatment induced the formation of root hairs (Masucci and Schiefelbein, 1994). The mutant *pip5k3*, which has a mutation in the phosphatidylinositol-(4,5)-bisphosphate-generating enzyme and which is localized in the plasma membrane, has very short root hairs with a normal distribution density (Kusano *et al.*, 2008). Therefore, few mutants with hairless roots and a non-pleiotropic phenotype under normal and stress conditions are available for *Arabidopsis*.

Studies on the calcium-binding protein, PCaP2, in the plasma membrane revealed the involvement of PCaP2 in root hair development as a signal transducer. During the study, overexpression of a 23 amino acid peptide in the N-terminal region of PCaP2 (N23) caused by the root hair-specific *EXPANSIN A7* promoter had the effect of suppressing root hair emergence and elongation (Kato *et al.*, 2013). In this study, line NR23 (NR23; *n*o *r*oot hair line that expresses N*23*), a root hair-less mutant of *Arabidopsis*, was used to investigate the physiological significance of root hairs. It was found that NR23 did not produce root hairs, even under phosphate (Pi)-deficient conditions or with ethylene treatment, which typically stimulate the production of root hairs (Pitts *et al.*, 1998; Dolan, 2001; Péret *et al.*, 2011; Chandrika *et al.*, 2013). In NR23, root hair cells have been specifically altered in such a way that the small 23 residues peptide of PCaP2 is expressed under the control of the root hair-specific promoter. Cell specificity has been demonstrated by expression of this peptide linked to green fluorescent protein (Kato *et al.*, 2013). In addition, it has been demonstrated that PCaP2 is not a transcription factor and its effect is restricted to the late phase of intracellular signalling. Based on these characteristics and the results of the phenotypic analysis, the effect found in NR23 is considered to be restricted to the production of root hair cells (Kato *et al.*, 2013).

The physiological significance of root hairs has been examined extensively (Gilroy and Jones, 2000; Minorsky, 2002; Libault *et al.*, 2010). Although some studies have shown that root hairs do not participate in root anchoring or the uptake of minerals such as Fe (Moog *et al.*, 1995; Schmidt *et al.*, 2000; Bayle *et al.*, 2011), these findings may have been attributed to differences in mutant lines or experimental procedures. It was considered that NR23 is well suited for examining the physiological significance of the root hair-less condition in *Arabidopsis*. Therefore, the capacity for the absorption of water and specific mineral nutrients, growth, and fruit production of NR23 were examined. In addition, the role of root hairs in root anchoring was also analysed using NR23. Here the phenotypic properties of NR23 are described, focusing on the potential disadvantages of the root hair-less condition, as well as the characteristics and molecular properties of NR23 grown under Pi-deficient conditions.

## Materials and methods

### Plant materials and growth conditions

*Arabidopsis thaliana* strain Col-0 was used as the WT. The seeds were surface-sterilized and germinated on sterile gel plates containing Murashige and Skoog (MS) salt, 2.5mM MES-KOH (pH 5.7), 1% (w/v) sucrose, and 0.8% Ina agar (Funakoshi). In an experiment to measure the proportion of plant roots that penetrated the gel to the total number of plants, a transparent gel of Gellan gum was used. In experiments on the effects of nutrient deficiency, seedlings were grown on plates containing 2.5mM MES-KOH (pH 5.7), 1% (w/v) sucrose, 1.2% Ina agar, and Hoagland medium, which lacked individual mineral such as Pi. The mineral components of the Ina agar and the Nacalai agar (Agar Purified) (Nacalai Tesque) were measured by inductively coupled plasma atomic emission spectroscopy (ICP-AES) (for media contents, see Supplementary Table S1 available at *JXB* online). For analyses of the effects of metal deficiency on seedling growth, agar containing low concentrations of the mineral under investigation was used. In the experiments to determine the numbers of branches and fruits, seedlings were germinated on agar plates for a few weeks, before being transplanted to pots containing vermiculite and grown with a 2000-fold diluted Hyponex (Hyponex Japan) solution. Furthermore, to assess growth in tap water or growth under water-stressed conditions, plants were grown on rockwool supplemented with tap water or a 2000-fold diluted Hyponex solution. Seedlings were grown at 22 °C under long-day conditions (16L8D photoperiod at 80–110 μmol m^–2^ s^–1^). In all experiments, seeds were incubated at 4 °C for 3 d in the dark before culture on medium.

### Measure of root length and width

The root length of seedlings was measured using image analysis software Lia32 (Yamamoto, 2000). The root width of seedlings was measured from photomicrographs taken with a stereomicroscope (Multi-Viewer system VB7010, Keyence). The free software package, ImageJ (http://rsbweb.nih.gov/ij/) was used to analyse pictures.

### Production of transgenic plants

The NR23 line (*A. thaliana*, strain Col-0), which expresses the first to the 23rd residues of the N-terminal region (N23 region) under the control of the root hair-specific promoter *EXPANSIN A7*, was prepared as described previously (Kato *et al.*, 2013). After selection with kanamycin, T_4_ plants were used for physiological experiments. F_1_ plants that contain both N23 and PIP5K3–yellow fluorescent protein (YFP)-coding genes were made by crossing the N23 line #6–9 with the transgenic lines 4 and 31 expressing PIP5K3–YFP under the control of the *PIP5K3* promoter (Kato *et al.*, 2013). Double homozygous F_3_ seeds were selected from the heterozygous F_1_ seeds with antibiotics and named #6–9 complementation 1 (line 4) and 2 (line 31), respectively. Seeds of the *cpc try* double knockout mutant were a kind gift of Dr Takuji Wada (Hiroshima University, Japan). A *pip5K3 pip5K4* double knockout mutant was prepared by crossing *pip5k3* and *pip5k4* (Kusano *et al.*, 2008).

### Determination of plant tissue mineral contents

*Arabidopsis* seedlings were grown on rockwool blocks or plates of Hoagland medium, which were set vertically. After 14–42 d, shoots and roots were collected from seedlings, washed three times in MilliQ water, and then dried at 70 °C for 48h. Dried tissues (10–50mg) were digested with pure concentrated HNO_3_ for 25min at 130 °C using Teflon vessels (Ethos-1600, Sorisole, Italy). The mineral contents of the seedlings were then determined by an ICP-AES (IRIS ICAP, Nippon Jarrell Ash) as described (Kawachi *et al.*, 2009).

### Growth under nutrient-limited conditions

Seedlings were grown on Hoagland medium lacking or containing reduced levels of a specific element (e.g. Pi). The concentrations of each element in each modified medium were Cu 0 μM, Ca 0.15mM, N 0.5mM, K 0.33mM, Fe 0.25 μM, Mn 0 μM, B 0 μM, Mg 0mM, Zn 0 μM, or P 0 μM. After 14–6 d, shoot and/or root fresh weight, root length, and root diameter were measured. The mineral contents of seedlings grown under these conditions were determined by ICP analysis.

### Measurement of absorbed water

Seedlings were grown on vertically set MS plates for 14 d and then seedling roots were set into wells of a 24-well plate. Into each well was poured 2.5ml of 2000-fold diluted Hyponex which was then overlaid with 0.5ml of liquid paraffin (Nacalai Tesque) to prevent evaporation of water from the wells. Seedling position was fixed by a plastic sheet, which had a small hole to support the shoot and to prevent the shoots from coming into contact with the paraffin (see Fig. 5). The volume of the remaining Hyponex solution was then measured after the seedlings were incubated in a culture room at 22 °C under long-day conditions.

### Growth under water-stressed conditions

Plants were grown in rockwool pots supplemented with 2000-fold diluted Hyponex solution for 2 weeks. The Hyponex solution was then removed and growth was observed under water-stressed conditions for a week. After removing the Hyponex solution, the diameter of the leaf area shown as a white dotted circle (see Fig. 6) was measured every day and the growth of Col-0 and NR23 was compared. In addition, the fresh weight of the shoots was also measured after 1 week.

### Number of branches, fruits, and seeds produced under normal conditions

Seedlings germinated and grown for 3 weeks on MS medium were transplanted to pots containing vermiculite and cultured in 2000-fold diluted Hyponex solution in a culture room (22 °C) for ~3 months. After the plants withered, branch number, fruit number, and seed number were measured. Average seed weight was measured using 200 seeds.

### Measurement of the proportion of roots that entered the gel

Seedlings were germinated on MS medium containing Gellan gum at the concentrations shown in Fig. 8. After 14 d, the percentage of roots that penetrated the gel was measured. Shoot fresh weight was measured at different Gellan gum concentrations.

### Acid phosphatase assay

Seeds were germinated in vertical gels so that roots could grow on the gel surface. To determine the total activity of acid phosphatases, roots from each seedling were submerged in 2ml of assay medium for 15min and activity was determined by measuring the absorbance at 550nm. The assay medium contained 50mM trisodium citrate buffer (pH 5.6), 2.5mM Fast Red TR (Sigma-Aldrich), and 3.4mM 1-naphthyl phosphate, which is an artificial substrate (Dinkelaker and Marschner, 1992).

### Quantification of malic and citric acids released from roots

Plants were grown for 3 weeks in Hoagland medium under normal and Pi-deficient conditions. Roots of five plants were submerged in water for 3h and then aliquots of the water samples were subjected to quantification of malic and citric acids using F-kits (Roche Applied Sciences), which are based on NADH-linked enzyme reaction. The F-kit for malate contained glutamic acid, NAD, glutamate-oxaloacetate transaminase, and malate dehydrogenase. The F-kit for citrate contained NADH, malate dehydrogenase, lactate dehydrogenase, and citrate lyase. After incubation at 25 °C for 30min, absorbance at 340nm was measured to determine the acid contents.

### Peptide preparation for LC-MS analysis

The crude membrane fractions were prepared from roots of 14-day-old seedlings. Roots were homogenized in 0.25M sorbitol, 50mM TRIS-acetate (pH 7.5), 1mM ethylene glycol bis(2-aminoethyl ether)tetraacetic acid, 20 μM *p*-(amidinophenyl)methanesulphonyl fluoride hydrochloride, 1% (w/v) polyvinyl pyrolidone, and 2mM dithiothreitol (DTT). The homogenates were centrifuged at 1000 *g* at 4 °C for 20min, and the supernatants were centrifuged at 5500 *g* at 4 °C for 15min. The obtained supernatants were centrifuged at 99 000 *g* at 4 °C for 60min. The pellets were dissolved in 0.5M triethylammoium bicarbonate (pH 8.5) and 0.1% SDS (Fukao *et al.*, 2011). The protein concentration was determined by the Bradford method (Bio-Rad). Three replicates were prepared from three independent experiments of seedlings grown separately.

The proteins in the crude membrane fractions were separated on a 12.5% SDS gel, which was stained using Flamingo Fluorescent Gel Stain (Bio-Rad). The gel slice isolated from each lane was cut into four pieces. Each gel piece was washed twice with 60% (v/v) acetonitrile/50mM ammonium bicarbonate for 10min at 25 °C. The gel pieces were treated with 10mM DTT/50mM ammonium bicarbonate for 45min at 56 °C to reduce proteins, and then with 55mM iodoacetamide/50mM ammonium bicarbonate for 30min at 25 °C in the dark to alkylate proteins. The treated gels were washed twice with 60% (v/v) acetonitrile/50mM ammonium bicarbonate for 10min at 25 °C, and then dried in a vacuum concentrator. To the dried gels were added 10ng μl^–1^ trypsin (Trypsin Gold, Mass Spec Grade, Promega) in 50mM ammonium bicarbonate. The gels were rehydrated for 5min at 25 °C, covered with 50mM ammonium bicarbonate, and incubated overnight at 37 °C. The incubated solution was transferred to a new tube. The gel pieces were treated with 50% (v/v) acetonitrile/0.2% (v/v) formic acid for 10min at 25 °C. The digested peptides were then pooled, dried in a Speed Vac for 2–4h, and then dissolved in 20 μl of 5% (v/v) acetonitrile/0.1% (v/v) formic acid. The digested peptides were filtered using Millipore Ultrafree-MC centrifugal filters (PVDF 0.45 μm, Millipore) at 12 000 *g* for 15min and used for liquid chromatography–mass spectrometry (LC-MS) analysis.

### Mass spectrometric analysis and database search

The peptides were loaded on the column (100 μm internal diameter, 15cm length; L-Column, CERI) using a Paradigm MS4 HPLC pump (Bruker) and HTC-PAL Autosampler (CTC Analytics), and then eluted using a gradient of 5–45% (v/v) acetonitrile in 0.1% (v/v) formic acid for 26min. The eluted peptides were introduced into an LTQ-Orbitrap XL mass spectrometer (Thermo Scientific) with a flow rate of 500 nl min^–1^ and a spray voltage of 2.0kV. The range of the MS scan was *m/z* 450–1500 (Fukao *et al.*, 2011). The three largest peaks were subjected to MS/MS analysis. MS/MS spectra were analysed using the MASCOT server (version 2.4) in house (Perkins *et al.*, 1999) (http://www.matrixscience.com/) and compared with proteins registered in TAIR10. The following Mascot search parameters were used: threshold of the ion score cut-off, 0.05; peptide tolerance, 10 ppm; MS/MS tolerance, 0.5 Da; and peptide charge, 2+ or 3+. The search was also set to allow one missed cleavage by trypsin, carbamidomethylation modification of cysteine residues, and variable oxidation modification of methionine residues.

### Quantification of mRNA of Pi transporters

Total RNA fractions were prepared from 14-day-old roots of Col-0 and NR23 grown under normal and Pi-deficient conditions using a QIA shredder and RNeasy Mini kit (Qiagen). RNA (1 μg) was converted into cDNA using an iScript-cDNA synthesis kit (Bio-Rad). Real-time reverse transcription–PCR (RT–PCR) analysis was performed with a Thermal Cycler Dice™ Real Time System TP800 (Takara) using a SyBR Green real-time PCR master mix (Toyobo). The primer sets used for the real-time RT–PCR were GGTGACAAACTCGGACGGAA (Fw) and AACCTGAAGAAGCAAAGGGTGG (Rv) for a subfamily of *PHT1* (phosphate transporters 1) and GGTAACATTGTGCTCAGTGGTGG (Fw) and CACGACCTTAATCTTCATGCTGC (Rv) for *Actin8*. Relative mRNA contents were normalized to the *Actin8* transcript, because Actin8 showed no change in proteomics analyses (data not shown).

## Results

### Suppression of growth and accumulation of minerals in NR23 under nutrient-limited conditions

**Fig. 1.**
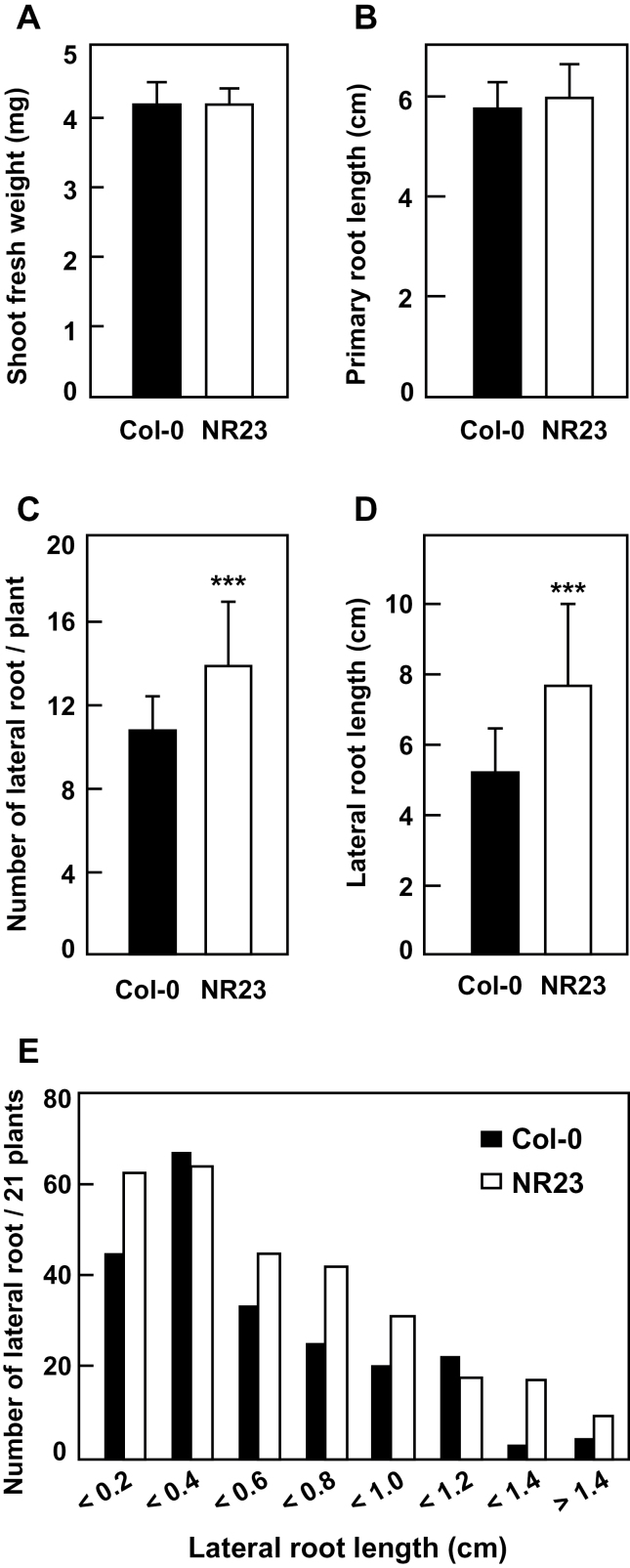
NR23 grew as well as Col-0 under normal conditions. The WT (Col-0) and NR23 were germinated under sterile conditions on 1× MS medium containing 1% sucrose. Plants were grown in a culture chamber at 22 °C under long-day conditions in vertical plates. Eleven-day-old seedlings were subjected to measurements of shoot fresh weight (A), primary root length (B), number of lateral roots per plant (C), and lateral root length per plant (D). The distribution of lateral root length in each range is shown in E. Error bars show ± SD (*n*≥21). Asterisks indicate a statistical difference (***P<0.005 by two-sided Student *t*-test).

Col-0 and NR23 were germinated on 1× MS medium or Hoagland medium in vertical plates. The fresh shoot weight and primary root lengths of NR23 were similar to those of Col-0 (Fig.1A, B), implying that NR23 grew normally under nutrient-rich conditions. However, compared with Col-0, the number and length of lateral roots in NR23 were 22% and 34% higher, respectively (Fig. 1C–E). This increase in the number and length of lateral roots may compensate for the loss of root hairs.

**Fig. 2.**
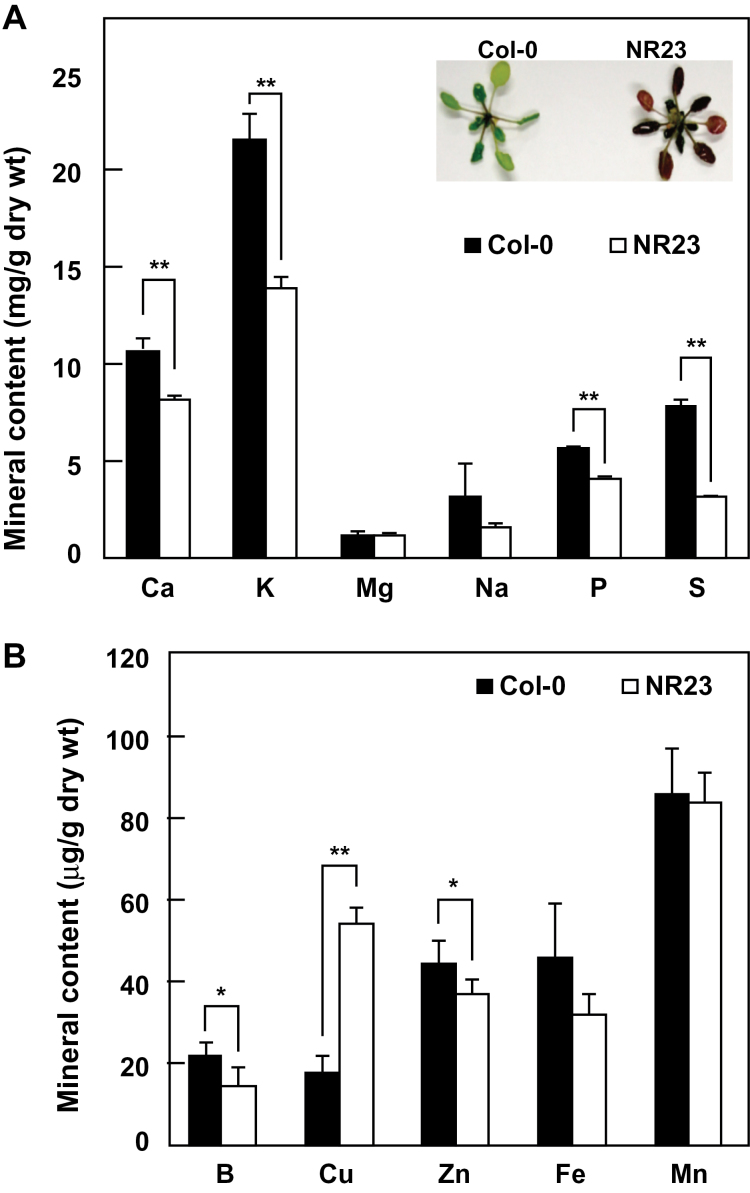
NR23 grew poorly and accumulated less minerals under nutrient-limited conditions. Col-0 and NR23 were grown with tap water on rockwool blocks in a culture room at 22 °C under long-day conditions for 6 weeks. The contents of the major (A) and minor (B) nutrients in shoots were measured by ICP-AES. Typical plants were photographed and inserted (A). Values are means ±SD; *n*=5. Asterisks indicate a statistical difference relative to Col-0 (*P<0.05; **P<0.01).

To examine the growth of NR23 under nutrient-limited conditions, Col-0 and NR23 were grown in rockwool pots with tap water. After 6 weeks, anthocyanin accumulation was greater in the shoots of NR23 than in Col-0 (Fig. 2), suggesting that the plants were under physiological stress (for a review, see Chalker-Scott, 1999). When analysed by ICP-AES, the contents of several mineral elements (i.e. Ca, K, P, S, B, and Zn) were lower in NR23 than they were in Col-0.

**Fig. 3.**
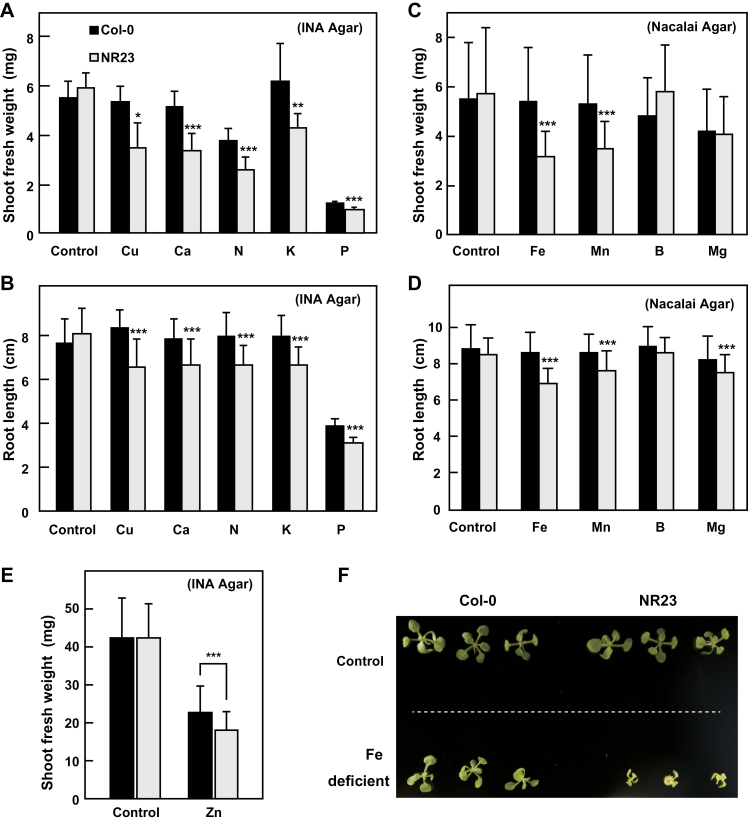
Growth of NR23 was poor under mineral-deficient conditions. Col-0 and NR23 were grown under sterile conditions on Hoagland medium containing 0.5% sucrose in vertical plates at 22 °C under long-day conditions. Deficiencies of Cu, Ca, N, K, P, and Zn were assayed using Ina agar, which contained low concentrations of a specific element (A, B, E). Nacalai agar was used for analyses of Fe, Mn, B, and Mg (C, D, F). Plants were subjected to measurements of fresh weight (A, C, E) and primary root length (B, D) at 14 d (A–D, F) or 26 d (E). Values are means ±SD; *n*≥30 (A–D), *n*=100 (E). Significant differences from Col-0 are indicated by asterisks (*P<0.05; **P<0.01, ***P<0.005). Typical seedlings grown under iron-deficient conditions were photographed (F).

To examine the contribution of root hairs to the absorption of nutrients and growth, NR23 were grown in medium deficient in a single mineral. Hoagland medium lacking specific elements (Cu, Ca, N, K, Fe, Mn, B, Mg, Zn, or P) was prepared, and was then used as the growth medium. Agar products produced by two different manufacturers were used for the experiments because the mineral contents of the agars differed markedly (Supplementary Table S1 at *JXB* online). When plants were germinated on medium without Cu, Ca, N, K, Fe, Mn, or P, shoot fresh weight and the root length of NR23 were lower than those of Col-0 (Fig. 3A–D). A typical example of NR23 grown under Fe-deficient conditions is shown in Fig. 3F. When plants were germinated and grown in medium lacking Zn, shoots of NR23 was significantly lighter than that of Col-0 although there was no difference in the fresh weight between Col-0 and NR23 in normal medium (Fig. 3E). In addition, roots of NR23 grown in medium containing low levels of nitrogen were small in diameter (Supplementary Fig. S1).

**Fig. 4.**
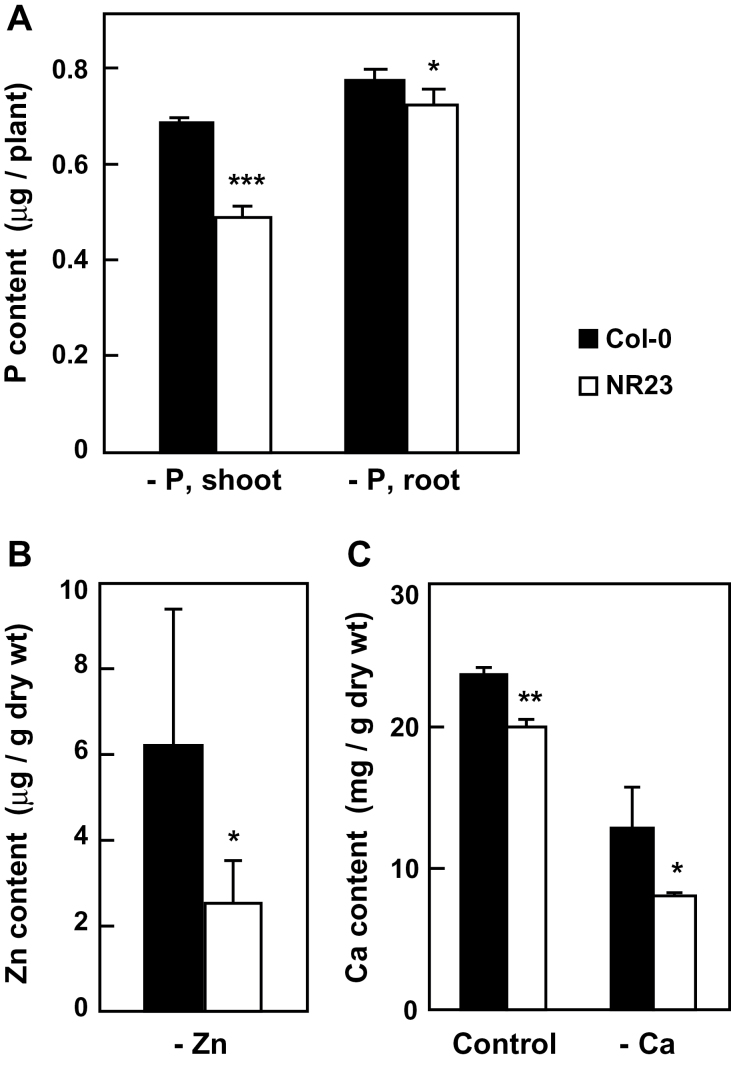
The metal content of NR23 grown under mineral-deficient conditions was low. Col-0 and NR23 were grown on Hoagland medium containing 0.5% sucrose and low concentrations of P (A), Zn (B), or Ca (C). After 14 d, the metal content in shoots (A–C) and roots (A) was measured by ICP-AES. Values are means ±SD; *n*≥4. *P<0.05, **P<0.01, ***P<0.005.

The lower growth observed in NR23 led to the determination of the contents of individual elements in the plant tissues by ICP analysis (Fig. 4). The contents of P, Zn, and Ca in NR23 grown under mineral-deficient conditions were markedly lower than those in Col-0. After growth on unmodified Hoagland medium, the contents of Ca, Fe, and Cu per dry weight in NR23 shoots were also lower than those in Col-0 (Supplementary Fig. S2 at *JXB* online). These results suggest that root hairs play an important role in absorbing P, Fe, Ca, Zn, and Cu. Under B-deficient conditions, growth of Col-0 and NR23 was normal (Fig. 3) and the content of B in plantlets of both lines that were grown in medium lacking B decreased by 66% compared with plantlets grown on normal medium (Supplementary Fig. S3). These findings suggest a minor role for root hairs in the absorption of B. For Mn, plant growth of NR23 was markedly suppressed under Mn-deficient conditions (Fig. 3), although there was no difference between Col-0 and NR23 in Mn content on a dry weight basis (Supplementary Fig. S2). Thus, the absolute amount of Mn in NR23 plants was markedly less than that in Col-0.

An NR23-related line (#2-1) that has short root hairs was germinated to examine whether or not root hair length is correlated to nutrient absorption. When plants were germinated on modified medium containing low concentrations of Cu, P, Ca, Fe, or Mn, shoot fresh weight and root length of the #2-1 line were intermediate compared with Col-0 and NR23 (Supplementary Fig. S4 at *JXB* online). The results showed that mineral absorption by roots was correlated to the root hair length and that decreased contents of these minerals in NR23 were due to it being root hair-less and not due to the other genes becoming dysfunctional by DNA insertion.

### NR23 was defective in water absorption and drought tolerance

**Fig. 5.**
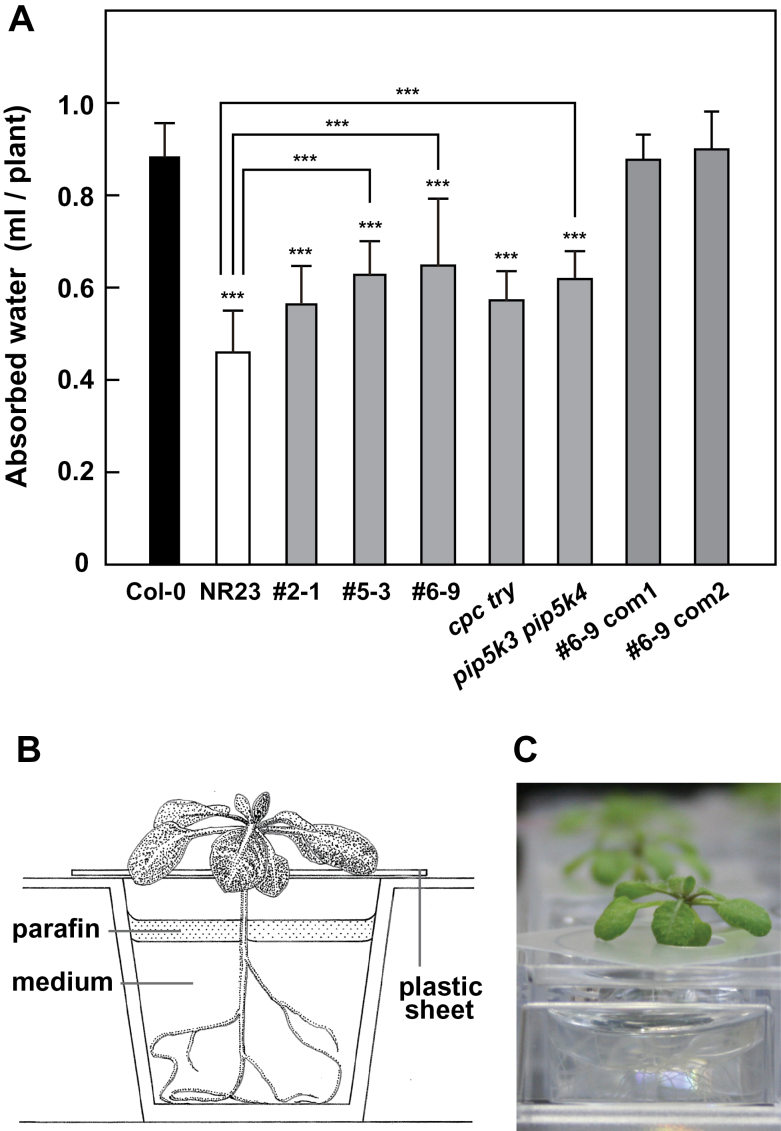
NR23 and related lines absorbed less water. (A) Col-0, NR23, and NR23-related lines (#2-1, #5-3, and #6-9), *cpc try*, *pip5k3 pip5k4*, and two complementation lines of #6-9 (#6-9 com1, #6-9 com2) were grown on 1× MS medium containing 1% sucrose for 14 d. The seedlings were transferred to wells of a 24-well-plate and incubated for 65h at 22 °C. The volume of water absorbed by each plant is shown. No loss of water was observed in wells without seedlings. Values are means ±SD; *n*>6. ***P<0.005. (B) Schematic model of water absorption assay. Each well was supplied with 2.5ml of a 2000-fold diluted Hyponex solution. After setting each plantlet on the well using a thin plastic sheet, 0.5ml of liquid paraffin was loaded onto the medium to avoid evaporation of water from the well. (C) Photograph after water absorption assay. Seedlings appeared healthy, even after the assay.

The capacity for water absorption was measured using 14-day-old seedlings of Col-0, NR23, short root hair NR23-related lines (#2-1, #5-3, and #6–9) (Kato *et al.*, 2013), *cpc try*, and *pip5k3 pip5k4*. Water absorbed by NR23 after 65h was 53% of that absorbed by Col-0 (Fig. 5). The values for #5-3, #6–9, and *pip5k3 pip5k4* were intermediate between those of Col-0 and NR23, while those for #2-1 and *cpc try* were slightly higher than that for NR23. Furthermore, the water absorption ability was clearly recovered in complementation lines of #6–9 with *PIP5K3*, whose root hairs were recovered as reported previously (Kato *et al.*, 2013). These results indicated that the longer root hairs facilitated the absorption of water. In the experiments, no water was lost from the assay wells not containing seedlings, which confirmed that the decrease in water was caused by the seedlings. Seedlings grew well during the assay, as shown in Fig. 5C. Furthermore, no difference was observed in the root length and leaf size of Col-0 and the other lines under the assay conditions.

**Fig. 6.**
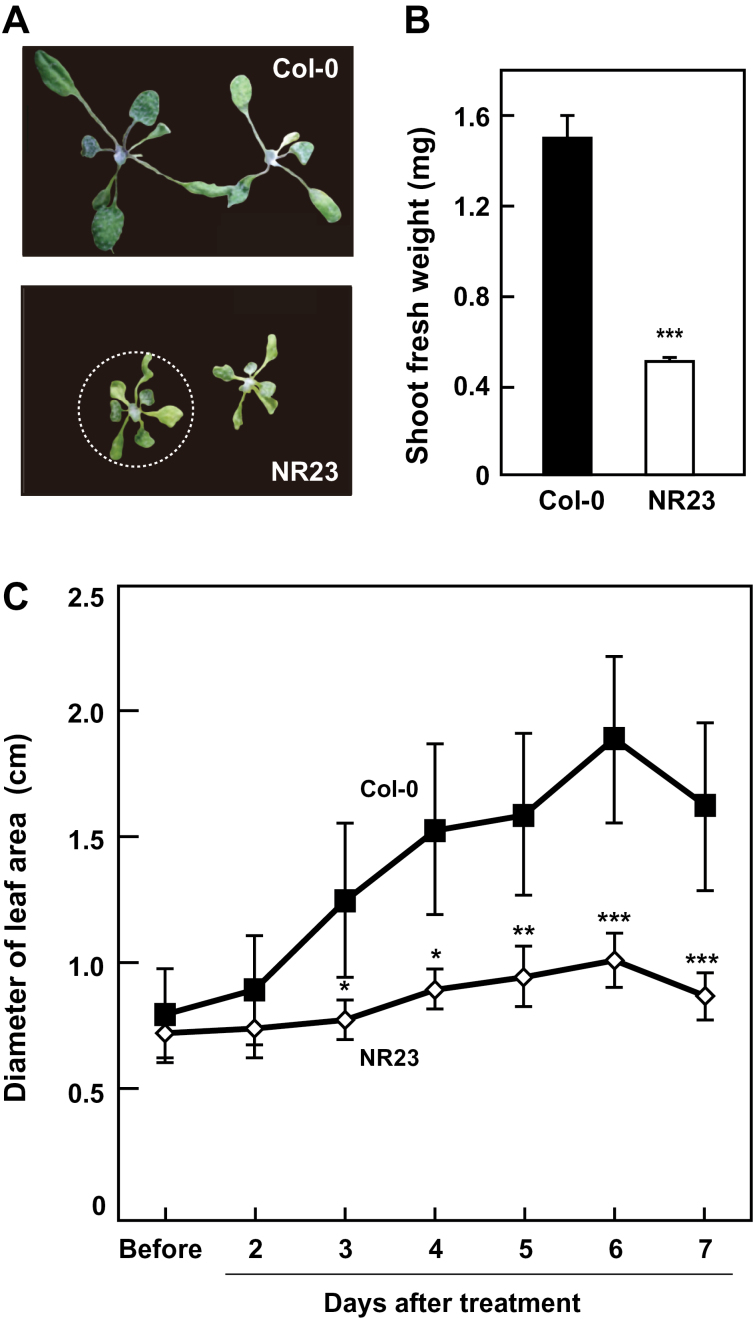
NR23 was less tolerant to drought. Col-0 and NR23 were grown on rockwool supplemented with a 2000-fold diluted Hyponex solution. After 14 d, water was withheld for an additional 7 d. Plants were grown in a culture room at 22 °C under long-day conditions. After the drought treatment, shoots were photographed (A) and weighed (B). Five replicates (eight seedlings) were averaged, and the SD is shown. (C) The maximal diameters (shown by the white dotted circle in A) of the same rosettes were measured daily. Values are means ±SD; *n*=4. *P<0.05, **P<0.01, ***P<0.005.

Col-0 and NR23 were grown on rockwool supplemented with a Hyponex solution. After 14 d, seedlings were not watered for another 7 d. After this water-stressed treatment, shoots were photographed and weighed (Fig. 6). The shoot fresh weight of NR23 was ~30% of that of Col-0. The maximal diameters of the rosette leaf area were measured every day. The diameter of the rosette leaf area of Col-0 increased constantly until 6 d after the onset of water-stressed conditions. On the other hand, NR23 showed a slight increase in leaf area. The rate of growth of NR23 was 25% of that of Col-0; leaf growth was stopped in both Col-0 and NR23 on day 6 (Fig. 6C). These results suggest that, compared with Col-0, NR23 is less tolerant of drought stress.

### NR23 was less adaptive to heat and salinity

The tolerance of NR23 to high temperatures was determined, and it was found to be in the range of natural, physiological conditions. The shoot fresh weight and chlorophyll content of NR23 were lower than those of Col-0 when grown at 22 °C for 12 d and then at 30 °C for 8 d (Supplementary Fig. S5 at *JXB* online). Col-0 grew better at 30 °C than at 22 °C, but no such enhancement was observed in NR23 at 30 °C. It was then examined whether the early stage of germination was sensitive to high temperatures, and it was found that growth at the germination stage of Col-0 and NR23 was severely suppressed at 30 °C (Supplementary Fig. S5D, E at *JXB* online). No difference was observed in shoot fresh weight and root length between Col-0 and NR23 grown under constant temperatures of 22 °C or 30 °C. However, when seedlings germinated and grown for 7 d at 22 °C were transferred to 30 °C and grown for a further 7 d, growth of NR23 was markedly suppressed compared with Col-0. The results indicated that growth of both lines was comparable at high temperatures, but that NR23 was more sensitive to a temperature shift from 22 °C to 30 °C. In other words, root hairs play a key role in short-term adaptation to high temperatures.

To compare salt tolerance, Col-0 and NR23 were grown in 1× MS medium with or without 50mM NaCl for 18 d. Growth of both Col-0 and NR23, but especially NR23, was inhibited on medium containing 50mM NaCl (Supplementary Fig. S6 at *JXB* online). The shoot fresh weight and root length of NR23 were ~20% lower than those of Col-0, suggesting that NR23 was less tolerant to salt stress.

### Reduction of numbers of branches and fruits in NR23 under normal conditions

**Fig. 7.**
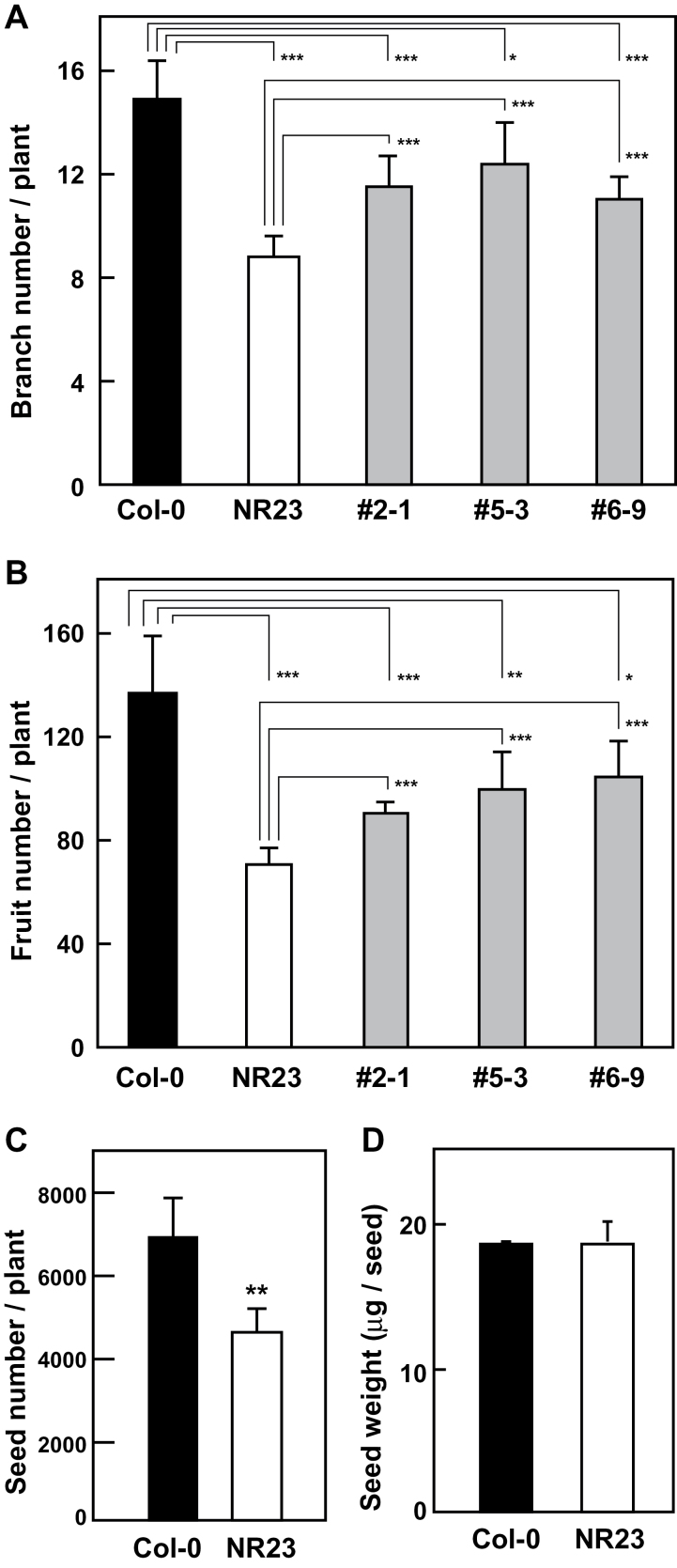
NR23 produced few branches and fruits under normal conditions. Col-0, NR23, and NR23-related lines were germinated on 1× MS medium containing 1% sucrose in vertical plates for 3 weeks. Seedlings were then transplanted to pots containing vermiculite and cultivated for 2 months at 22 °C under long-day conditions. Plants were watered with 2000-fold diluted Hyponex solution. After plants withered, branch number (A), fruit number (B), seed number (C), and seed weight (D) were measured. Values are means ±SD; *n*=4. *P<0.05, **P<0.01, ***P<0.005.

Col-0, NR23, and NR23-related lines were cultivated in pots containing vermiculite for 2 months, and the numbers of branches, fruits, and seeds, and seed weight were measured. Compared with Col-0, there were considerably fewer branches, fruits, and seeds in NR23 (Fig. 7A–C), although the seed weight (Fig. 7D) and the germination rate (data not shown) of NR23 were the same as those of Col-0. Furthermore, the numbers of branches and fruits of NR23-related lines (#2-1, #5-3, and #6–9) were between those of Col-0 and NR23 (Fig. 7A, B). Interestingly, at the differentiation stage of the shoot meristem, the number of secondary shoots remained unchanged in NR23, but the number of the tertiary, quaternary, and younger shoots decreased considerably (Supplementary Fig. S7 at *JXB* online).

### Roots of NR23 did not penetrate the hard gel

**Fig. 8.**
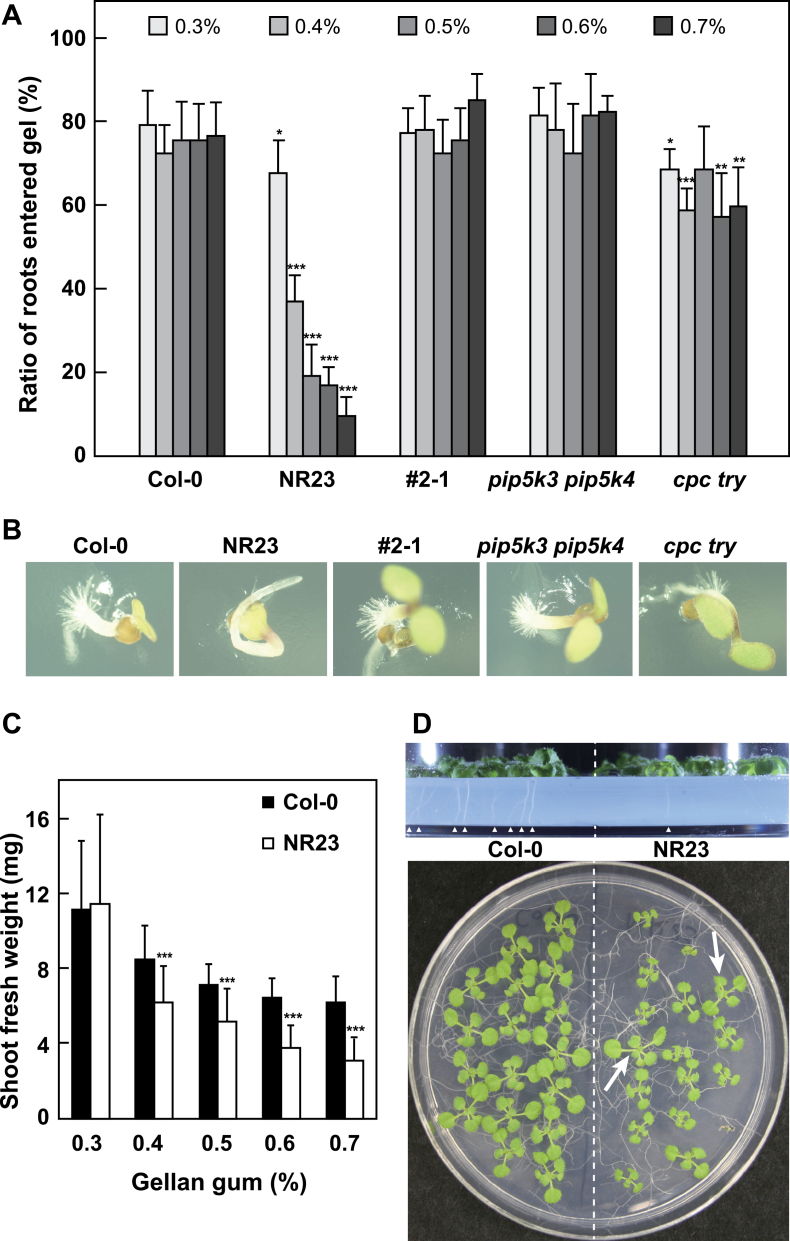
Roots of NR23 did not penetrate hard gels. Col-0, NR23, #2-1, *pip5k3 pip5k4*, and *cpc try* were grown on plates containing 1× MS medium and 1% sucrose, and hardened with Gellan gum at the concentrations indicated. (A) After 14 d, the percentage of plants whose roots penetrated the gel compared with the total number of plants measured. The results of six replicates were averaged, and the SD is shown. (B) Typical 2-day-old seedlings grown in 0.7% Gellan gum plates were photographed. Seedling roots except for NR23 penetrated the hard gel. (C) Shoot fresh weight was measured and compared with that of Col-0 at each concentration of Gellan gum. Values are means ±SD; *n*=34. ***P<0.005. (D) Photographs of Col-0 and NR23 grown for 2 weeks in 0.7% Gellan gum. Arrowheads indicate primary roots that penetrated the gel. Arrows in the lower photograph indicate NR23 seedlings whose roots penetrated the gel.

Col-0 and NR23 were grown in Gellan gum plates for 14 d and the percentages of roots that penetrated the gel were measured at each concentration of Gellan gum (Fig. 8). Roots of both Col-0 and NR23 easily penetrated the soft gels (Gellan gum, 0.3%). However, <50% and 9% of the roots of NR23 plants penetrated gels containing Gellan gum concentrations of 0.4% or 0.7%, respectively, whereas 87% of the roots of Col-0 plants penetrated even the 0.7% gel. Moreover, the fresh weight of shoots from both plant lines grown on harder gels was lower. Interestingly, the fresh weight of NR23 shoots was markedly lower than that of Col-0 at any of the gel concentrations exceeding 0.4%. The shoots of NR23, whose roots did not penetrate the gel, were smaller than those of seedlings which had roots that did penetrate the gel (Fig. 8D; lower panel, arrows).

As in Col-0, the roots of the other mutant plant lines, #2-1 and *pip5k3 pip5k4*, which had short root hairs, were able to penetrate the hard gels (Fig. 8A, B). Importantly, the roots of *cpc try*, which had root hairs at the shoot–root transit region, penetrated the gel, although the values were somewhat smaller than those of Col-0, #2-1, and *pip5k3 pip5k4*. The results suggest that root hairs in the transit region are important for roots to be able to penetrate the gel.

### Amounts of mRNA and protein of Pi transporters in NR23 were reduced under Pi-deficient conditions

Root hairs increase in number and length in WT plants under Pi-deficient conditions (Péret *et al.*, 2011), whereas no root hairs emerged in NR23 even under Pi-deficient conditions (Supplementary Fig. S8A at *JXB* online). During the study, several NR23-related lines were prepared and the line #2-1 was selected as being intermediate between Col-0 and NR23 (Kato *et al.*, 2013). The number and length of root hairs in line #2-1 increased under Pi-deficient conditions, even though the root hairs in line #2-1 are short under normal and Pi-deficient conditions.

**Fig. 9.**
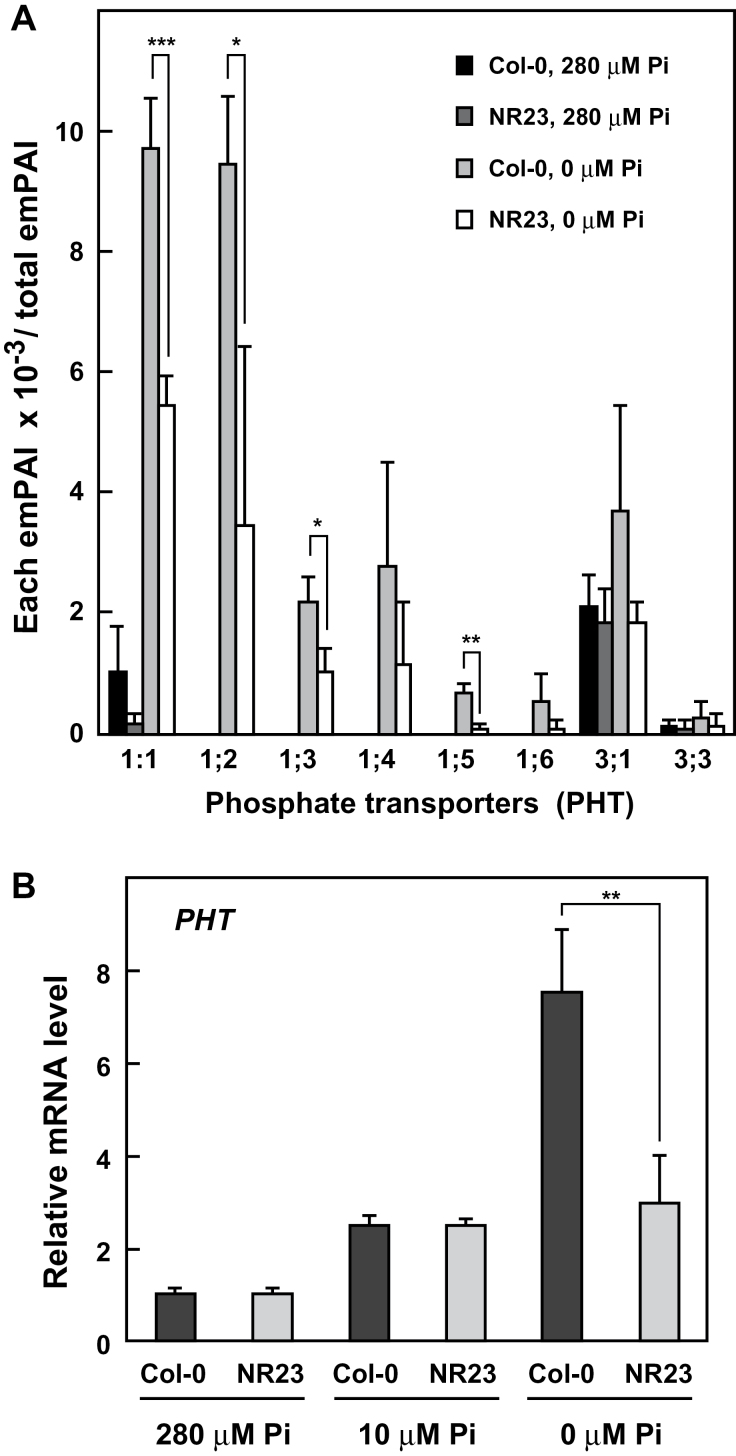
Contents of proteins and mRNAs of phosphate transporters were significantly lower in NR23 than in Col-0 under Pi-deficient conditions. Col-0 and NR23 were germinated and grown for 14 d in Hoagland medium containing 0, 10, or 280 μM phosphorus. (A) Crude membrane fractions were prepared from roots and used for protein MS analysis as described in the Materials and methods. Three replicates with >100 roots were averaged, and the SD is shown. Eight isoforms of phosphate transporters, PHT1;1, PHT1;2, PHT1;3, PHT1;4, PHT1;5, PHT1;6, PHT3;1, and PHT3;3, were detected. (B) Total RNA fractions were prepared from roots and used for real-time quantitative PCR. Four replicates with >100 roots were averaged and the SD is shown. *P<0.05, **P<0.01, ***P<0.005.

It was hypothesized that stimulation of root hair formation under Pi-deficient conditions may be related to the increase of Pi transporters on the root surface. To test this hypothesis, proteomic assays of crude membrane fractions prepared from roots of Col-0 and NR23 were conducted by MS analysis. Identified Pi transporters were shown in terms of the exponentially modified protein abundance index (emPAI; Ishihama *et al.*, 2005) values (Fig. 9A). In this assay system, only the product of a root hair-specific gene for *RHS13* (Won *et al.*, 2009; Bruex *et al.*, 2012) was detected in Col-0, but not in NR23, indicating that the membrane preparations provided information on the proteins closely related to root hairs. The amounts of all Pi transporters increased under the Pi-deficient condition. In particular, five transporters PHT1;2–PHT1;6 were undetectable in plants grown under normal conditions and were only detected in plants grown under Pi-deficient conditions. The increase in PHT1 proteins is consistent with previous observations in which PHT1 mRNAs increased under Pi-deficient conditions (Mudge *et al.*, 2002). Protein amounts of six Pi transporters (PHT1;1–PHT1;6) belonging to the PHT1 subfamily were increased under the Pi-deficient conditions, both in Col-0 and in NR23. However, in NR23 grown under Pi-deficient conditions, the amount of total mRNAs and corresponding protein levels belonging to the PHT1 subfamily was approximately half that in Col-0 (Fig. 9). Determining the transcript levels of each member of the PHT1 subfamily is currently difficult, especially for PHT1;1 and PHT1;2, because the base sequences are highly homologous among the members (public open database, The Arabidopsis Information Resource, http://www.arabidopsis.org/).

### Changes in protein profiles under Pi-deficient conditions in NR23

To examine the differences in protein profiles between Col-0 and NR23, proteomic analysis of crude membrane fractions prepared from roots was performed. The results showed marked changes in several proteins associated with Pi deficiency (Supplementary Fig. S9 at *JXB* online). Vacuolar membrane H^+^-pyrophosphatase (H^+^-PPase) was increased in both Col-0 and NR23 under Pi-deficient conditions. In Col-0, subunits A and C of vacuolar H^+^-ATPase (V-ATPase) were also increased under Pi-deficient conditions; the subunits showed similar changes in NR23, but the changes were not statistically significant. The marked increase in other subunits was also detected under Pi-deficient conditions.

The vacuolar membrane aquaporin, TIP1;2, occurred at the same levels in Col-0 and NR23, but increased markedly only in Col-0 under Pi-deficient conditions (Supplementary Fig. S9 at *JXB* online). The increase in TIP1;2 levels therefore seems to be closely related to the induction of root hair formation under Pi-deficient conditions. Marked changes in Pi deficiency were detected in other membrane proteins. Ammonium transporter 1;2, which is predominantly expressed in roots and is involved in ammonium uptake (Shelden *et al.*, 2001), was detected in Col-0 grown under Pi-deficient conditions, but not in NR23. Conversely, K^+^ channel β subunit 1 (KAB1) was markedly induced by Pi deficiency in NR23. KAB1 functions in the plasma membrane as a complex with AKT1 (*Arabidopsis* inwardly directed K^+^ channel) and is involved in the uptake of K^+^ into roots (Tang *et al.*, 1996; Lagarde *et al.*, 1996; Gierth *et al.*, 2005).

### Decrease of acid phosphatase and organic acids secreted from roots of NR23 under Pi-deficient conditions

**Fig. 10.**
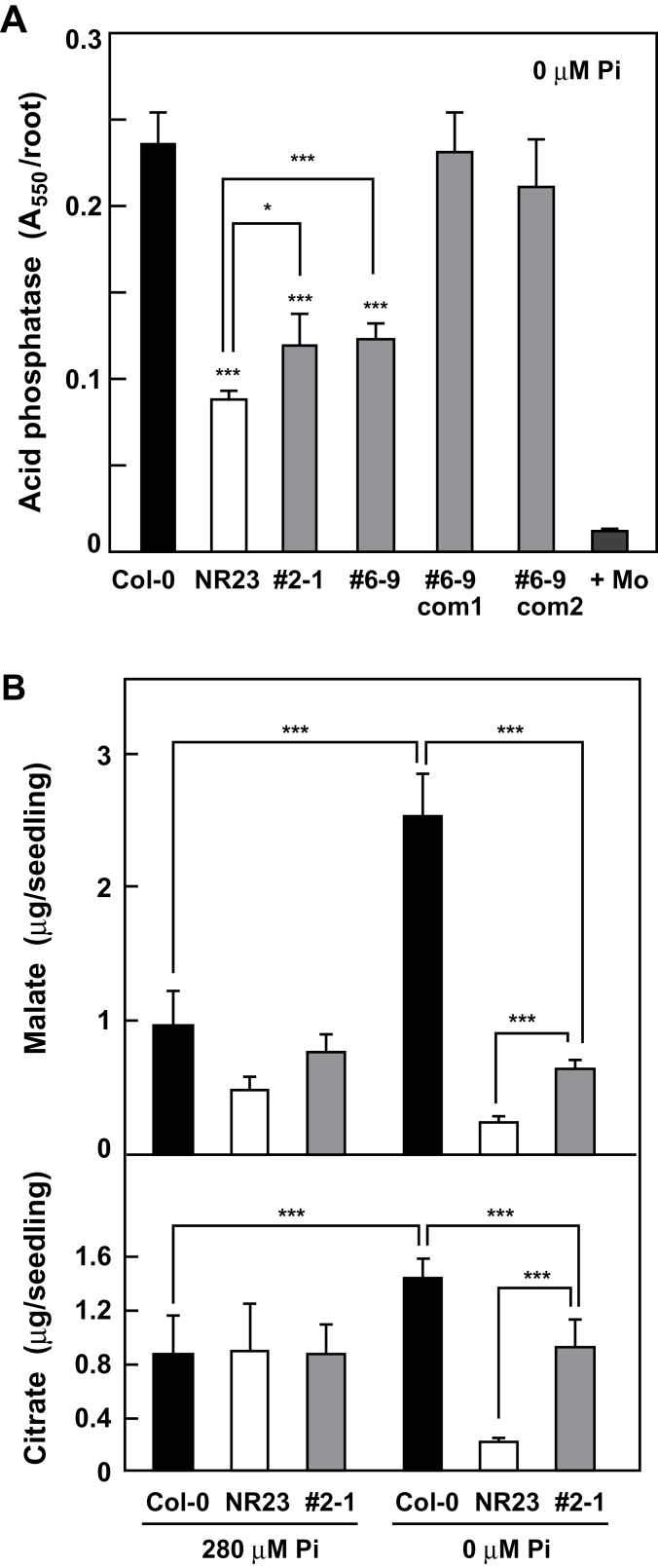
Secretion of acid phosphatases and organic acids was reduced in NR23 under phosphate-deficient conditions. (A) A root from each seedling of Col-0, NR23, NR23-related lines (#2-1 and #6-9), and complementation lines of #6-9 (com1 and com2) was submerged in 2ml of the reaction medium for 9min as described in the Materials and methods. It was confirmed that the activity was completely inhibited by molybdate at 1mM (+Mo), indicating that the detected activity was of the acid phosphatase. Values are means ±SD (*n*=5). *P<0.05. (B) Secretion of organic acids was reduced in NR23 under Pi-deficient conditions. Roots of 21-day-old seedlings were submerged in water for 3h and then aliquots of the water were used for quantification of malate and citrate. Values are means ±SD (*n*=5). ***P<0.005.

The differences in the amount of enzymes and organic acids secreted from the roots of Col-0 and NR23 were examined. The secreted enzymes were trapped on wet filter paper and assayed for acid phosphatases (Supplementary Fig. S10 at *JXB* online). Compared with roots grown at a normal Pi concentration of 280 μM, the intensity of red colour, which is an indicator of the activity of the secreted acid phosphatases, was higher in Col-0 and NR23 roots subjected to Pi-deficient conditions. The activity of the acid phosphatase secreted from NR23 roots was 38% that of Col-0 roots, even when NR23 was cultivated under Pi-deficient conditions (Fig. 10A). Furthermore, the values for #2-1 and #6–9 were intermediate between those of Col-0 and NR23. The acid phosphatase secreted from the #6–9 complementation lines was recovered to the level of Col-0. The results indicated that roots with longer root hairs secreted acid phosphatases.

Compared with normal conditions, the amount of malate secreted from Col-0 roots under Pi-deficient conditions increased to 2.6-fold (Fig. 10B). The amount of malate secreted by NR23 roots was half that of Col-0 under normal conditions and 9% of that secreted by Col-0 roots under Pi-deficient conditions. There was no difference in the amount of citrate between Col-0 and NR23 under normal conditions. Under Pi-deficient conditions, however, the amount of citrate secreted from Col-0 and NR23 roots increased and decreased significantly, respectively. Furthermore, it should be noted that the amounts of malate and citrate secreted from #2-1 were intermediate between those of Col-0 and NR23. The results suggest that the induction of organic acid secretion in response to Pi deficiency is highly dependent on the presence of root hairs.

## Discussion

Numerous studies have been conducted on the differentiation and elongation of root hairs at the molecular biological level (Datta *et al.*, 2011; Grebe, 2012; Kwasniewski *et al.*, 2013). Here the root hair-less *Arabidopsis* line, NR23, which was produced from the overexpression of the N-terminal region of PCaP2 (N23) in a root hair cell-specific manner (Kato *et al.*, 2013), was investigated. PCaP2 is a possible signal transducer, converting calcium signals to phosphatidylinositol signals on the plasma membrane of root hair cells (Kato *et al.*, 2013), and is considered to be essential for normal development and tip growth of root hair cells. It has been proposed that the loss of root hairs may result from problems with regulation of root hair cell elongation (Kato *et al.*, 2013). In support of this idea, the suppression of the phenotype caused by N23 was rescued by expression of PIP5K3 in F_1_ seedlings from a cross between an N23 line and a transgenic line expressing PIP5K3 under the control of the root hair cell-specific *PIP5K3* promoter (Kato *et al.*, 2013).

Several *Arabidopsis* lines have been reported to be root hair-less mutants. In order to clarify the property of root hairs of NR23, the formation and growth of root hairs of several of these lines are described. The mutant *cpc try* (Schellmann *et al.*, 2002) produced root hairs in the shoot–root transit region, but not in the maturation zone under normal conditions (Supplementary Fig. S8B at *JXB* online). In the experiments carried out here, *cpc try* produced normal root hairs in the maturation zones of primary and lateral roots under Pi-deficient conditions. The single gene mutant *cpc* (Wada *et al.*, 1997) has been reported to produce a morphological phenotype that is similar to that of *cpc try*. Another root hair-less line, *rhd6* (*r*oot *h*air *d*efective 6), has been reported to produce few root hairs under normal conditions, and more root hairs after treatment with an ethylene precursor (Masucci and Schiefelbein, 1994). The mutant *pip5k3* has remarkably short root hairs with a normal distribution density (Kusano *et al.*, 2008). In contrast to these mutant lines, NR23 produced no root hairs on any parts of the root under normal or Pi-deficient conditions (Supplementary Fig. S8A), or after ethylene treatment (data not shown). Furthermore, NR23 grew as well as Col-0 under normal nutritional conditions (Fig. 1). If growth of a root hair-less line is defective under normal conditions, then it is very difficult to evaluate the effect of various stresses on the line. Based on the characteristics of these root hair-less mutants, it was concluded that NR23 is well suited for use as a root hair-less mutant under any conditions.

Compared with Col-0, NR23 had numerous long lateral roots under normal conditions, possibly to compensate for being root hair-less. However, with regard to water and mineral absorption by the roots, as discussed below, the increase in the number of lateral roots was not able to compensate for the loss of root hairs in NR23. Here disadvantages associated with the loss of root hairs in NR23 are described as are the changes in molecular characteristics, especially membrane transporters, of NR23 grown under Pi-deficient conditions.

### Key role of root hairs in water absorption and their role in tolerance to drought, heat adaptation, and salinity

The present study showed that the water absorption is correlated with the presence of root hairs and their length. Compared with Col-0, NR23 showed a 47% reduction in water absorption (Fig. 5). Further, NR23-related short root hair lines, such as #2-1 and #6–9, showed water absorption characteristics that were intermediate between those of Col-0 and NR23. The reduced ability for water absorption of #6–9 was recovered to the level of Col-0 when the *PIP5K3* gene was introduced into #6–9 under its own promoter (Fig. 5). In contrast to root hairs, there was no difference in root length and leaf size between Col-0 and NR23, which implies that the quantity of absorbed water is dependent upon the quantity of root hairs. Based on these results, it was considered that a substantial proportion of water was absorbed through root hairs and the remaining water was incorporated through the other epidermal cells of roots.

The reduced water absorption of NR23 was reflected in the high sensitivity of NR23 to drought. Col-0 grew well for several days, even under water-limited conditions, but the growth of NR23 was strongly suppressed under the same conditions (Fig. 6). It might be difficult for NR23 to absorb sufficient quantities of water through primary and lateral roots alone in rockwool pots that were not irrigated. In contrast, the root hairs of Col-0 absorbed water that had been retained by the rockwool, even in the absence of irrigation. These observations indicate that this is one of the key physiological functions of root hairs in soil.

The capacity of NR23 to adapt to high temperatures was significantly lower than that of Col-0, as shown by shoot fresh weight (Supplementary Fig. S5 at *JXB* online). This poor heat adaptation might be due to the relatively lower ability to absorb water. Col-0 maintained an optimal water balance between absorption and transpiration, even at 30 °C. However, in contrast to Col-0, NR23 was unable to absorb sufficient quantities of water for active transpiration when temperatures suddenly increased. Furthermore, growth in Col-0 and NR23 was suppressed under high salinity conditions, but more so in NR23 (Supplementary Fig. S6). This relatively lower salt tolerance in NR23 may be explained by the reduction in water absorption associated with the root hair-less condition, because absorption of water is essential for diluting salt in root cells and for distributing ions to different tissues. Also, it should be noted that NR23 is considered to have a low capacity for K^+^ accumulation (Figs 2, 3), because the ratio of K^+^ to Na^+^ in tissues is tightly related to salt tolerance of plants (Maathuis and Amtmann, 1999). Indeed, the low ratio of K^+^ to Na^+^ may explain the relatively higher salt sensitivity of NR23.

### Root hairs are essential for penetrating hard gels

A previous study showed that root hairs do not contribute to anchoring the roots (Bailey *et al.*, 2002). In that study, the pulling resistance of seedlings was determined and compared between the WT and the root hair-less mutant *rhd2-1*. In this study, the proportion of seedlings whose roots were capable of penetrating soft and hard gels was determined (Fig. 8). Less than 50% of the roots of NR23 were capable of penetrating 0.4% Gellan gum gels, with this proportion decreasing to only 9% of plants being able to penetrate the 0.7% gel; however, Col-0 showed 87% penetration in the same gels. NR23 exhibited normal gravitropism in these experiments (data not shown). In the other lines, *cpc try* only had root hairs in the shoot–root transit region, and two lines #2-1 and *pip5k3 pip5k4* had short root hairs. These three lines were able to penetrate the hard gels, although the extent of penetration in *cpc try* was less than it was in #2-1 and *pip5k3 pip5k4*. It was therefore concluded that root hairs in the transit region play a key role in the capacity of roots to penetrate gels together with root hairs in the mature and elongating root zones. In soil conditions, root hairs of crops may play an important role in the roots clinging to soil particles. This role might be reflected in the efficiency of absorption of mineral nutrients and water when grown in soils, although the present study was carried out in agar gels.

### Role of root hairs in mineral nutrient uptake

Some controversy exists over the role of root hairs in the uptake of several mineral nutrients (Gilroy and Jones, 2000; Minorsky, 2002; Libault *et al.*, 2010). For example, the contribution of root hairs to the uptake of Fe and Si has been reported to be minor. Mineral uptake in NR23 was examined under two different conditions. When seeds were grown on rockwool without nutrients, the Ca, K, P, S, B, and Zn contents of NR23 seedlings were lower than those in Col-0 (Fig. 2). Furthermore, NR23 did not grow well in media lacking N, K, Ca, P, Fe, Mn, Zn, or Cu (Figs 3, 4; Supplementary Fig. S4 at *JXB* online), indicating that root hairs play an important role in the uptake of at least these minerals; of these, N, K, Ca, and P are macronutrients. These observations corroborate those of previous reports. For example, deficiency of P, Fe, and Mn has been reported to induce formation of root hairs in plants (Schmidt *et al.*, 2000; López-Bucio *et al.*, 2003; Yang *et al.*, 2008; Chandrika *et al.*, 2013). Regarding the absorption of N, three nitrate transporters (Huang *et al.*, 1996, 1999; Krouk *et al.*, 2006; Wirth *et al.*, 2007) and an ammonium transporter (Engineer *et al.*, 2007) are expressed in root epidermal cells and root hairs. IRT1 is expressed in epidermal and cortical cells and is involved in the absorption of Fe (Vert *et al.*, 2002). The Cu transporters, COPT1 and COPT2, are involved in Cu uptake from the soil (Sancenón *et al.*, 2004). The loss of root hair7s may thus result in a reduction of these mineral transporters. Although information on the genes and/or proteins associated with the membrane transporters expressed in root hairs is available (Libault *et al.*, 2010), neither IRT1 nor COPT2 has been described, primarily due to difficulties associated with isolating root hair cells together with all of the young trichoblasts and elongating and mature root hairs. Further study on the root hair-specific membrane transporters is therefore necessary in order to understand root hair-specific uptake of minerals and water.

Contradictory results were obtained for B in two of the experiments performed in this study. The content of B in NR23 shoots grown on rockwool without minerals was low (Fig. 2), but the growth of NR23 under B-deficient conditions was normal (Fig. 3). The B contents were markedly decreased in Col-0 and NR23, and amounts were equivalent under B-deficient conditions (Supplementary Fig. S3 at *JXB* online). It is likely that the minimum levels of B were maintained in NR23 roots and that the reduction of B in NR23 roots grown on rockwool might be a secondary effect caused by a deficiency in most of the essential minerals. B is passively incorporated into epidermal cells and then actively translocated in the stele by BOR1 (borate transporter 1) located in the pericycle (Takano *et al.*, 2002). Consequently, the contribution of root hairs to the incorporation of B may be negligible.

### Marked reduction in Pi transporters and in secretion of acid phosphatases and organic acids in NR23 under Pi-deficient conditions

A marked increase in root hair development in response to a Pi deficit has been extensively reported in a variety of plants (Foehse and Jungk, 1983; Gahoonia *et al.*, 2001; Ma *et al.*, 2001). Here, the molecular properties of several membrane transporters whose roles in root hairs were revealed in this study are discussed. The present study showed that the amounts of several Pi transporters increased under Pi-deficient conditions. Among these, PHT1;1, PHT1;2, PHT1;3, and PHT1;4 are all expressed in root epidermal cells and are thought to be involved in Pi uptake as high-affinity Pi transporters (Mudge *et al.*, 2002; Shin *et al.*, 2004; Bayle *et al.*, 2011). The induced levels of PHT1s in NR23 were, at most, half of that observed in Col-0 under Pi-deficient conditions (Fig. 9). It is thus possible that these four PHT1s may be predominantly expressed in root hairs where they would probably play a key role in the acquisition of Pi under Pi-limited conditions. The total mRNA levels in all nine members of the PHT1 family were increased 2.5-fold in both Col-0 and NR23, corroborating a recent microarray study in which PHT1;1 and PHT1;2 increased 3-fold in *Arabidopsis* roots in 10 μM medium (Chandrika *et al.*, 2013). Interestingly, at 10 μM Pi, the total mRNA levels of PHT1s were increased 3-fold in Col-0 and NR23, with an additional marked increase observed in Col-0 but not in NR23 at 0 μM Pi (Fig. 9B). It is therefore considered that induction of the four PHT1s in transcription and translation under Pi-depleted conditions occurs mainly in the root hairs.

It was found that several other membrane proteins were increased under Pi-deficient conditions. For example, AMT1;2, an ammonium transporter, was markedly induced in Col-0 but not in NR23, indicating that root hair-specific expression of AMT1;2 occurred (Supplementary Fig. S9 at *JXB* online). Vacuolar H^+^-PPase, subunits A and C of V-ATPase, and aquaporin TIP1;2 were also significantly increased in Col-0 at 0 μM Pi. Conversely, in NR23, the increments in the V-ATPase subunits A and C were relatively low compared with levels in Col-0, implying that quantitative up-regulation of V-ATPase is essential for the adaptation of roots (and root hair formation) to Pi deficiency. H^+^-PPase protein amounts have also been reported to increase under Pi-deficient conditions (Yang *et al.*, 2007). As with V-ATPase subunits, increments of H^+^-PPase in NR23 were slightly lower than that in Col-0. Aquaporin TIP1;2, one of the major TIP members (Boursiac *et al.*, 2005), was increased at 0 μM Pi in Col-0 but not in NR23. From their biochemical roles (Martinoia *et al.*, 2007), it is speculated that enhancement of root hair formation requires vacuolar proton pumps and aquaporin to support the rapid enlargement of root hair cells. In addition, there is another reason why H^+^-PPase and V-ATPase are required for production of Pi from pyrophosphate and ATP, respectively, under Pi-limited conditions.

Roots are known to secrete acid phosphatases, which hydrolyse organic Pi compounds and release inorganic Pi (Neumann *et al.*, 2000; Li *et al.*, 2002). In Col-0 and NR23, secretion of acid phosphatases was less under normal conditions and enhanced under Pi-deficient conditions (Supplementary Fig. S10 at *JXB* online), and the enhanced level of secreted acid phosphatases was markedly reduced in NR23 compared with Col-0. The reduced amount of acid phosphtases in #6-9 with short root hairs was complemented by expression of *PIP5K3* under its own promoter. Recovery in secretion of acid phosphatases and water absorption (Fig. 5) in the two complementation lines may exclude the possibility that the observed phenotypes of NR23 are caused by unknown genetic changes occurring in the transgenic process. It was therefore concluded that some of the acid phosphatases were secreted from root hairs. Several isoforms of the 29 members of the acid phosphatase family, some of which are secreted and others of which are localized in vacuoles, are markedly increased under conditions of Pi deprivation (Li *et al.*, 2002); for example, the transcription of a vacuolar-type AtPAP26 is enhanced under Pi-deficient conditions (Hurley *et al.*, 2010). However, further clarification of the various isoform(s) secreted by root hairs is still necessary.

Malic and citric acids are major organic acids secreted from roots (Neumann *et al.*, 2000). These acids acidify the soil and promote the release of Pi from immobilized soil compounds, such as Fe-Pi and Al-Pi (Gerke, 1994; Jones, 1998). The released Pi is then incorporated into roots. Although the Col-0 secreted malate and citrate under Pi-deficient conditions (Fig. 10), the amounts of secreted acids from NR23 were markedly reduced, even under Pi-deficient conditions. The mutant #2-1, which has short root hairs, secreted acids at levels that were intermediate between Col-0 and NR23. The results clearly indicated the essential role that root hairs play in the secretion of malate and citrate under Pi-deficient conditions. These results are consistent with previous studies of barley cultivars with short root hairs and without root hairs under Pi-deficient conditions. These cultivars with abnormal root hairs secreted less acid phosphatases and absorbed a lower amount of Pi under Pi-deficient conditions compared with a cultivar with normal length root hairs (Gahoonia *et al.*, 2001; Gahoonia and Nielsen, 2003).

Although information on root hair-specific organic acid transporters is not available, there is a report that AtALMT3 (aluminium-induced malate transporter 3; malate exporter) is expressed in root hairs and transcription is enhanced under Pi-deficient conditions (Maruyama *et al.*, 2011). In conclusion, root hairs were shown to play a role in the secretion of organic acids, but determination of root hair-specific transporters in acid secretion remains to be resolved.

### Reduction in the number of branches and fruits in NR23 under normal conditions

The reduction in water absorption and nutrient uptake observed in NR23 resulted in a decrease in the numbers of branches and fruits compared with Col-0 (Fig. 7; Supplementary Fig. S7 at *JXB* online). NR23-related lines, such as #2-1, produced an intermediate number of branches and fruits. Interestingly, the numbers of secondary shoots in NR23 and Col-0 were similar, but the number of tertiary, quaternary, and greater branches was remarkably decreased in NR23. Thus, the most marked changes in the physiology of NR23 becomes apparent in the shoots in the latter stages of shoot growth, but not at the initial differentiation of the primary shoot.

In conclusion, *Arabidopsis* seedlings with no root hairs exhibit a reduced capacity for water absorption and the uptake of mineral nutrients P, Fe, Ca, Zn, Cu, and K. For Mn and N, plant growth of NR23 was markedly suppressed under Mn- or N-deficient conditions (Fig. 3; Supplementary Fig. S1 at *JXB* online). It is therefore considered that, in addition to the six aforementioned minerals, root hairs are also involved in the uptake of Mn and N.

The present study also showed that the absence of root hairs affected seedling growth and resulted in a reduction in the number of fruits and seeds. Root hair-less plants exhibited a lower tolerance to drought and salinity, and a reduced capacity for heat adaptation and secretion of organic acids and acid phosphatases, all of which may be critical for plant survival in adverse environmental conditions. Furthermore, plant roots that were not capable of penetrating the gel may not survive under natural conditions. The multifaceted approach of this study revealed that root hairs have a variety of functions. Further studies are required to identify the various aquaporins, mineral transporters, exporters of organic acids, and acid phosphatases that are specific to root hairs, how many hours or days they are functional in the life cycle of the root hair, and how the numbers and lengths of root hairs are regulated under conditions of physiological stress.

## Supplementary data

Supplementary data are available at *JXB* online.

Figure S1. Root diameter of NR23 was small under nitrogen-limited conditions.

Figure S2. Content of several metals in NR23 differed from that in Col-0 grown in normal medium.

Figure S3. Borate accumulation was not reduced in NR23.

Figure S4. An NR23-related line showed poor growth under metal-deficient conditions.

Figure S5. NR23 was less tolerant to heat adaptation.

Figure S6. NR23 was less tolerant to salinity.

Figure S7. NR23 generated fewer shoots.

Figure S8. Root hair numbers of Col-0, NR23, and its related mutant lines under Pi-deficient conditions.

Figure S9. Protein amounts of aquaporins, H^+^-pyrophosphatase, vacuolar H^+^-ATPase, ammonium transporter1;2, and potassium channel beta subunit 1 changed markedly under Pi-deficient conditions.

Figure S10. Secretion of acid phosphatases was reduced in NR23.

Table S1. Contents of elements in Ina and Nacalai agars determined by ICP-AES.

Supplementary Data

## Abbreviations:

ICP-AES: inductively coupled plasma atomic emission spectroscopy
N23: 23 amino acid peptide in the N-terminal region of PCaP2
NR23: no root hair line that expresses N23
PCaP2: plasma membrane-associated Ca^2+^-binding protein-2
