# Supplementary material for: Characteristics of a root hair-less line of Arabidopsis thaliana under physiological stresses
Source: J Exp Bot. 2014 Feb 5;65(6):1497–512. doi: 10.1093/jxb/eru014 (PMC3967087; doi:10.1093/jxb/eru014)
Supplement: Supplementary Data [file supp_65_6_1497__index.html]

Characteristics of a root hair-less line of Arabidopsis thaliana under physiological stresses — Characteristics of a root hair-less line of Arabidopsis thaliana under physiological stresses — Supplementary Data 

# Characteristics of a root hair-less line of *Arabidopsis thaliana* under physiological stresses

## Supplementary Data

Data files

**Files in this Data Supplement:**

- Supplementary Data - Supplementary Data
