# Supplementary material for: Characteristics of a root hair-less line of Arabidopsis thaliana under physiological stresses
Source: J Exp Bot. 2014 Feb 5;65(6):1497–512. doi: 10.1093/jxb/eru014 (PMC3967087; doi:10.1093/jxb/eru014)
Supplement: Supplementary Data [file supp_eru014_jexbot106773_file001.pdf]

Characteristics of a root hair-less line of *Arabidopsis thaliana* under physiological stresses

Natsuki Tanaka[1], Mariko Kato[2], Rie Tomioka[1], Rie Kurata[3], Yoichiro Fukao[3], Takashi Aoyama[2], and Masayoshi Maeshima[1,*]

Methods for experiments shown in supplemental information

Plant materials and growth conditions

Seeds of WT and mutant lines were germinated on sterile gel plates containing MS salt, 2.5 mM MES-KOH (pH 5.7), 1% (w/v) sucrose, and 0.8% Ina agar (Funakoshi). In experiments on the effects of nutrient deficiency, seedlings were grown on plates containing 2.5 mM MES-KOH (pH 5.7), 1% (w/v) sucrose, 1.2% Ina agar, and Hoagland medium, which lacked individual minerals such as Pi. For analyses of metal deficiency on seedling growth, agar containing low concentrations of the mineral under investigation was used. In the experiments examining the effect of high temperatures on plant growth, and to determine the numbers of branches, seedlings were germinated on vertical agar plates for several days (experiments for high temperatures) and 3 weeks (experiments for branch numbers), before being transplanted to pots containing vermiculite and grown with a 2000-fold diluted Hyponex (Hyponex Japan) solution. Seedlings were grown at 22 °C under long-day conditions (16L8D photoperiod at 80–110 μmol m^{-2} s^{-1}). In all experiments, seeds were incubated at 4 °C for 3 d in the dark and before culture on medium.

Assessing tolerance to high temperatures and high salinity

Seedlings of Arabidopsis strains Col-0 and NR23 were germinated in 1× MS medium at 22 °C for 9 d were transferred to pots containing vermiculite and grown for 3 d. Seedlings were then grown at 22 or 30 °C for another 8 d to examine the tolerance of seedlings to high temperatures. To assess salt tolerance, seedlings were germinated on MS medium with or without 50 mM NaCl for 18 d.

Determination of mineral contents in agars

The mineral components of the Ina agar (Funakoshi) and the Nacalai agar (Agar Purified) (Nacalai Tesque) were measured by inductively coupled plasma atomic emission spectroscopy (ICP-AES). Dried agars were digested with pure concentrated HNO_3 for 25 min at 130 °C using Teflon vessels (Ethos-1600, Sorisole, Italy). The mineral contents were then determined by an ICP-AES (IRIS ICAP, Nippon Jarrell Ash).

Chlorophyll quantification

Shoots (0.20 g fresh weight) were homogenized in 0.8 ml of acetone. The homogenates were centrifuged at 10,000*g* at 0 °C for 10 min and the obtained supernatants were made up to 2.0 ml using 80% acetone. Chlorophyll *a* and *b* contents (mg/ml) were determined based on absorbance of the extract at 663 and 645 nm using the following equations (Arnon, 1949).

Chlorophyll $a = 0.017\ A_{663} – 0.00259\ A_{645}$

Chlorophyll $b = 0.0229\ A_{645} – 0.00467\ A_{663}$

Acid phosphatase assay

Seeds were germinated in vertical gels so that roots could grow on the gel surfaces. Acid phosphatases secreted from roots were collected on filter paper wetted with the assay medium containing 50 mM trisodium citrate buffer, pH 5.6, 2.5 mM Fast Red TR (Sigma-Aldrich), 3.4 mM 1-naphthyl phosphate, which is an artificial substrate, according to Dinkelaker and Marschner (1992).

Mass spectrometric analysis and database search

Mass spectrometric analysis was conducted as described in the main text. The peptides were loaded on the column (L-Column, CERI) using a Paradigm MS4 HPLC pump and HTC-PAL Autosampler, and then eluted using a gradient of 5 to 45% (v/v) acetonitrile in 0.1% (v/v) formic acid for 26 min. The eluted peptides were introduced into an LTQ-Orbitrap XL mass spectrometer with a flow rate of 500 nl min^{-1} and a spray voltage of 2.0 kV. The range of the MS scan was m/z 450 to 1500. The three largest peaks were subjected to MS/MS analysis. MS/MS spectra were analyzed using the Mascot server (version 2.4) in house (Perkins et al., 1999) and compared with proteins registered in TAIR10. The following Mascot search parameters were used: threshold of the ion score cutoff, 0.05; peptide tolerance, 10 ppm; MS/MS tolerance, 0.5 D; and peptide charge, 2+ or 3+. The search was also set to allow one missed cleavage by trypsin, carbamidomethylation modification of cysteine residues, and variable oxidation modification of methionine residues.

References

Arnon DI. 1949. Copper enzymes in isolated chloroplasts: polyphenol oxidase in *Beta vulgaris*. *Plant Physiology* **24,** 1–15.

Dinkelaker B, Marschner H. 1992. In vivo demonstration of acid phosphatase activity in the rhizosphere of soil-grown plants. *Plant and Soil* **144,** 199–205.

Supplementary Table S1. Contents of elements in Ina and Nacalai agars determined by ICP-AES. Values are shown as averages with SD (n = 3).

		Al	Ca	K	Mg	Na	B	Cr	Fe	Mn	Zn	P	S	Si
INA		µg/g	mg/g	µg/g	µg/g	mg/g	µg/g	µg/g	µg/g	µg/g	µg/g	µg/g	mg/g	µg/g
Agar	Ave	14.1	1.63	13.0	661	0.20	76.7	0.64	27.6	14.4	0.20	9.99	1.53	116
	SD	0.9	0.00	1.7	5	0.00	0.4	0.01	3.8	4.9	0.07	0.87	0.02	8
Nacalai		µg/g	µg/g	µg/g	µg/g	mg/g	µg/g	µg/g	µg/g	µg/g	µg/g	µg/g	mg/g	µg/g
Agar	Ave	2.89	61.9	10.3	74.8	1.34	7.88	0.10	6.09	1.51	0.53	33.9	1.25	25.5
	SD	1.16	2.96	2.1	0.8	0.01	0.33	0.04	3.24	1.94	0.38	0.8	0.01	3.5

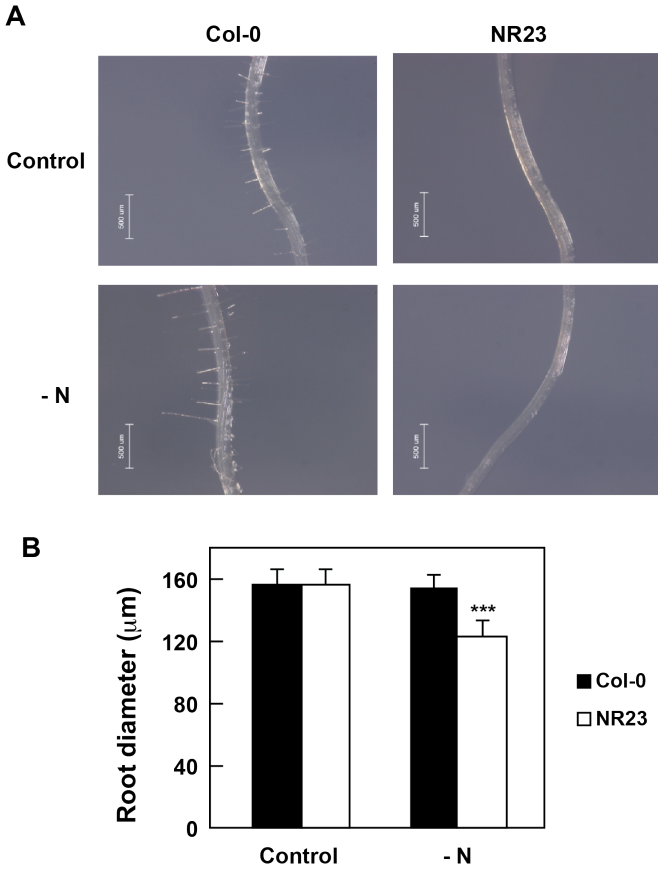

Fig. S1. Root diameter of NR23 was small under nitrogen-limited conditions. Col-0 and NR23 were grown in unmodified (Control) or nitrogen-limited (–N) Hoagland medium for 14 d. Roots were photographed (A) and their diameters on the photographs were measured using ImageJ software (B). Values are means ± SD (n =12). Asterisks indicate a statistical difference compared to Col-0 (***P < 0.005). Bars mean 0.5 mm (A).

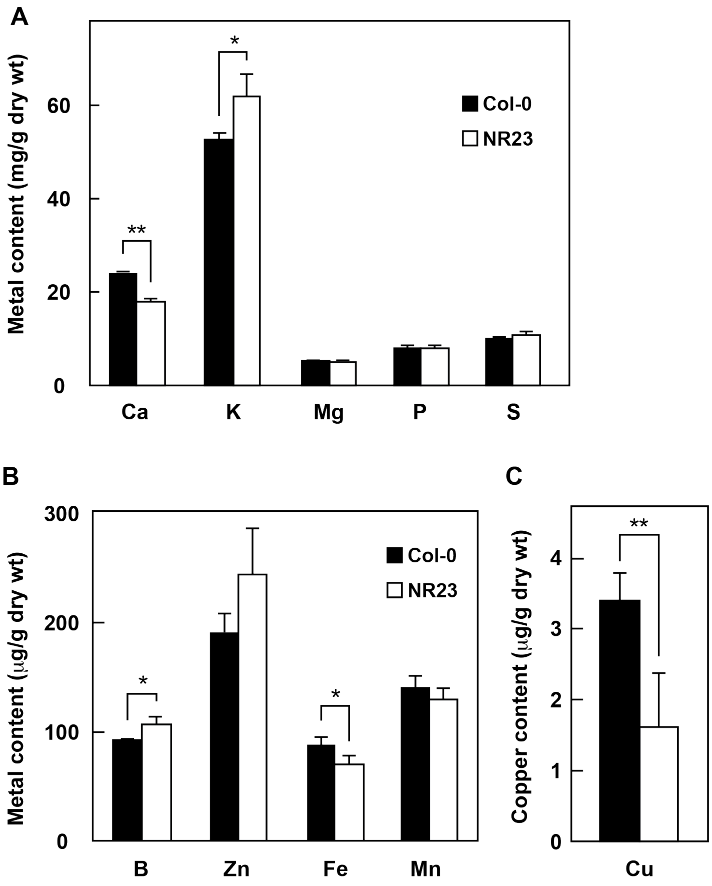

Fig. S2. Content of several metals in NR23 differed from Col-0 grown in normal medium. Col-0 and NR23 were grown in Hoagland medium containing 0.5% sucrose at 22 °C for 14 d under long-day conditions in vertical plates. The contents of elements in shoots were measured by ICP-AES. Major (A) and minor elements (B and C) are shown as means ± SD (*n* =5). **P* < 0.05, ***P* < 0.01.

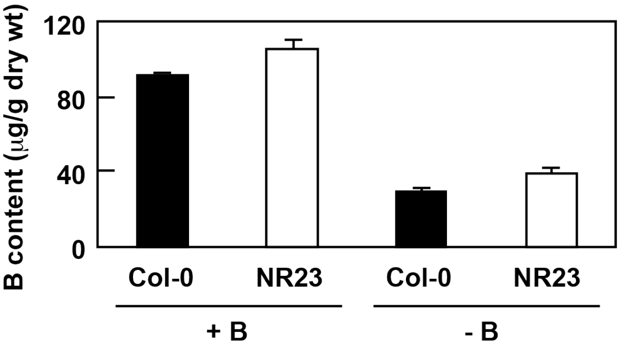

Fig. S3. Borate accumulation was not reduced in NR23. Col-0 and NR23 were grown on normal (+B) or boron-deficient (−B) Hoagland medium for 14 d, and the boron content of shoots was measured by ICP-AES. Results of four replicates were averaged and the SD is shown.

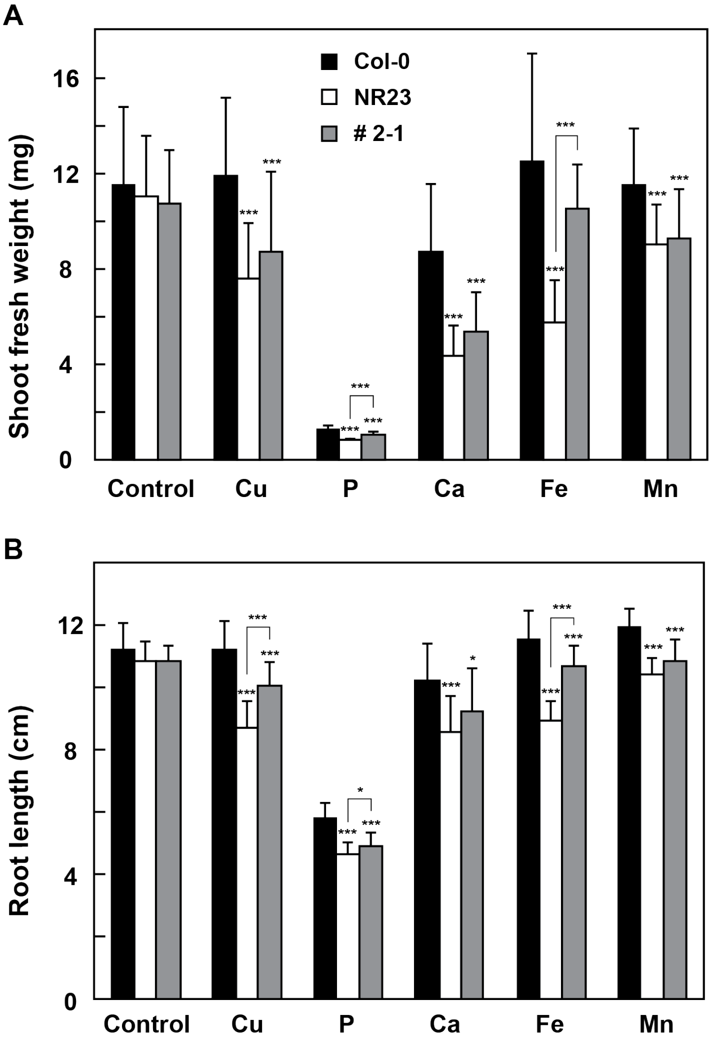

Fig. S4. NR23-related line showed poor growth under metal-deficient conditions. Col-0, NR23, and NR23-related line (#2-1) were grown in Hoagland medium deficient in one of the following metals: Cu, P, Ca, Fe, or Mn. Seedlings were grown in a culture chamber at 22 °C for 14 d under long-day conditions in vertical plates as described in the Materials and Methods. Fresh weight (A) and primary root length (B) were measured and are shown as the mean ± SD ($n \geq 20$). $^{*}P < 0.05$, $^{***}P < 0.005$.

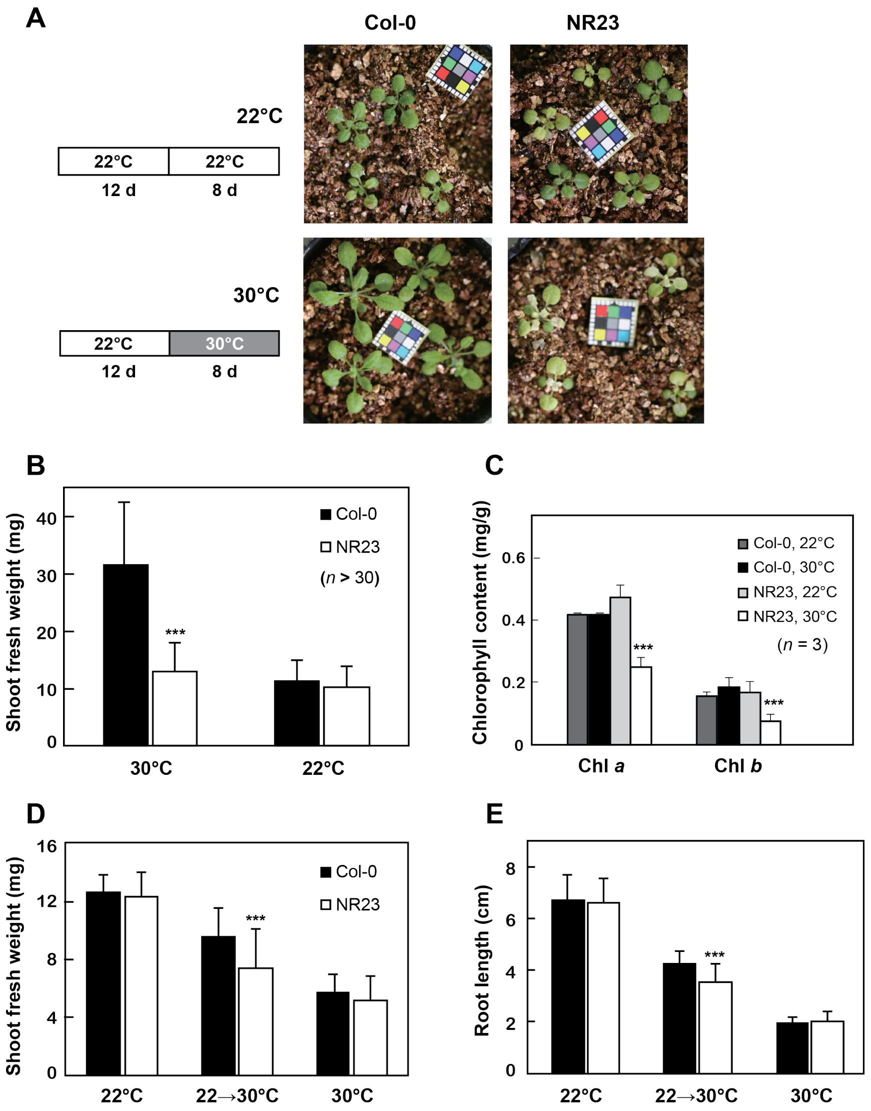

Fig. S5. NR23 was less tolerant to heat adaptation. (A–C) Seedlings of Col-0 and NR23 germinated in 1× MS medium at 22 °C for 9 d were transferred to pots containing vermiculite and grown 22 °C for 3 d, before being kept at 22 °C or transferred to 30 °C for a further 8 d (A). For estimates of color, each photograph included a color marker. (B) Shoot fresh weight is shown as the mean ± SD (*n* > 30). (C) Chlorophyll content in shoots was determined independently in triplicate and shown as the mean ± SD. (D and E) To examine the effect of high temperatures on seed germination, Col-0 and NR23 were grown in 1× MS medium under three temperature conditions: at 22°C for 14 d (22°C), at 22°C for 7 d followed by at 30°C for 7 d (22→30°C), or at 30°C for 14 d (30°C). Shoot fresh weight (D) and primary root length (E) are shown as mean ± SD (*n* =30). ***$P < 0.005$.

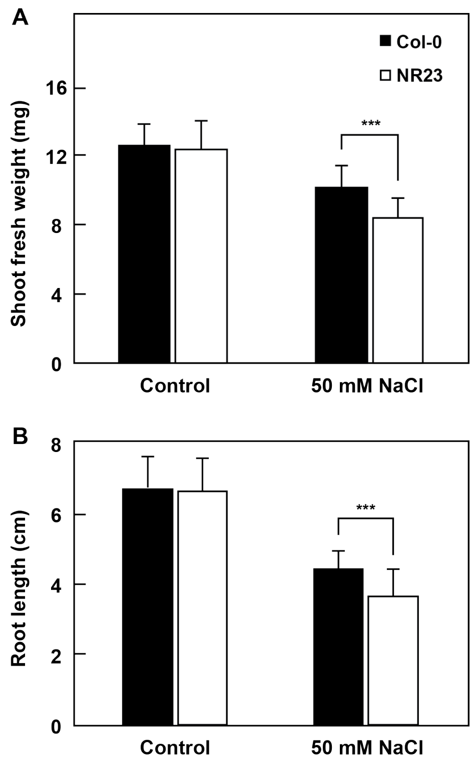

Fig. S6. NR23 was less tolerant to salinity. Col-0 and NR23 were grown in 1× MS medium with or without 50 mM NaCl for 18 d. (A) Shoot fresh weight was determined and shown as the mean ± SD. Eleven replicates (11 seedlings) were averaged. (B) Root length was determined and shown as the mean ± SD ($n \geq 90$). ***$P < 0.005$.

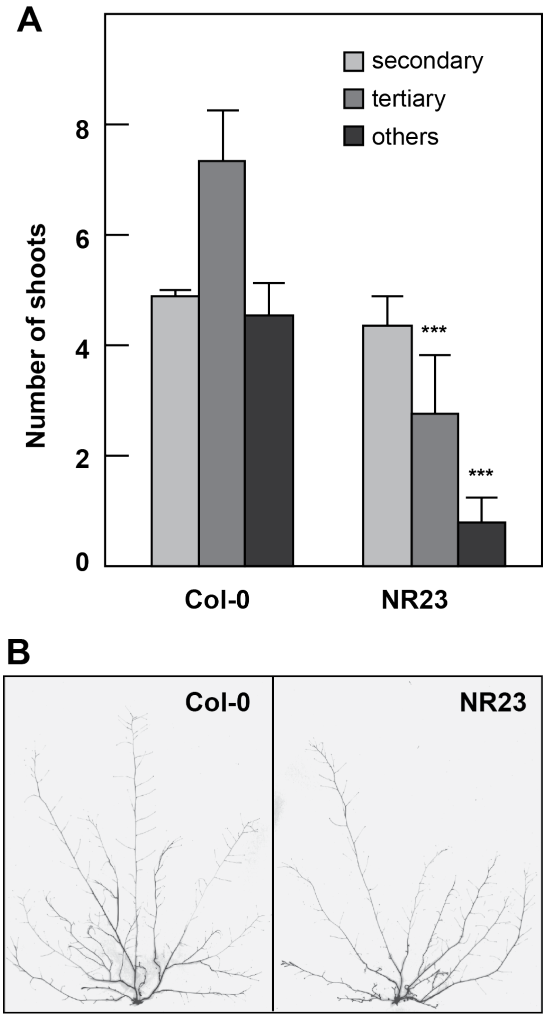

Fig. S7. NR23 generated fewer shoots. The numbers of shoots produced by mature Col-0 and NR23 plants were counted (A). Branches from the primary shoots (the longest branch) were regarded as secondary shoots. Branches from the secondary shoots were regarded as tertiary shoots. Further branches were regarded as other shoots. Values are means ± SD (n = 5). ***P < 0.005. Typical shoots of Col-0 and NR23 were photographed (B).

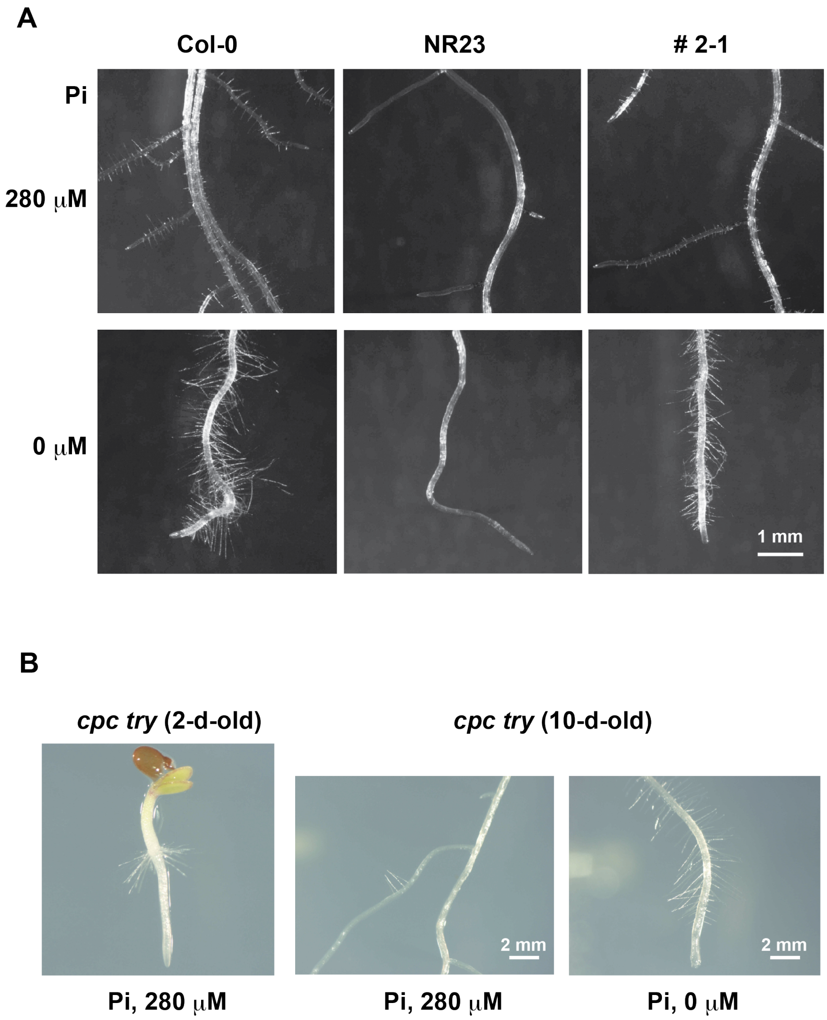

Fig. S8. Root hair number of Col-0, NR23, and its related mutant lines under Pi-deficient conditions. (A) Root hair numbers of Col-0 but not of NR23 increased under Pi-deficient conditions. Col-0, NR23, and NR23-related line (#2-1) were grown in unmodified (Pi, 280 μM) or Pi-deficient medium (Pi, 0 μM) in modified Hoagland solution. Roots of 14-day-old seedlings were photographed using a stereomicroscope. (B) Characteristics of root hairs of *cpc try*. A double mutant *cpc try* was grown in normal (280 μM Pi, left and middle panels) Hoagland medium or Pi-depleted medium (0 μM Pi, right). *cpc try* had root hairs only at the shoot-root transit region of 2-d-old seedlings under normal conditions. At 10 d, sporadic root hairs were observed in the maturation region in seedlings grown under the normal conditions, with many more root hairs in the elongating regions of the primary and lateral roots under Pi-deficient condition.

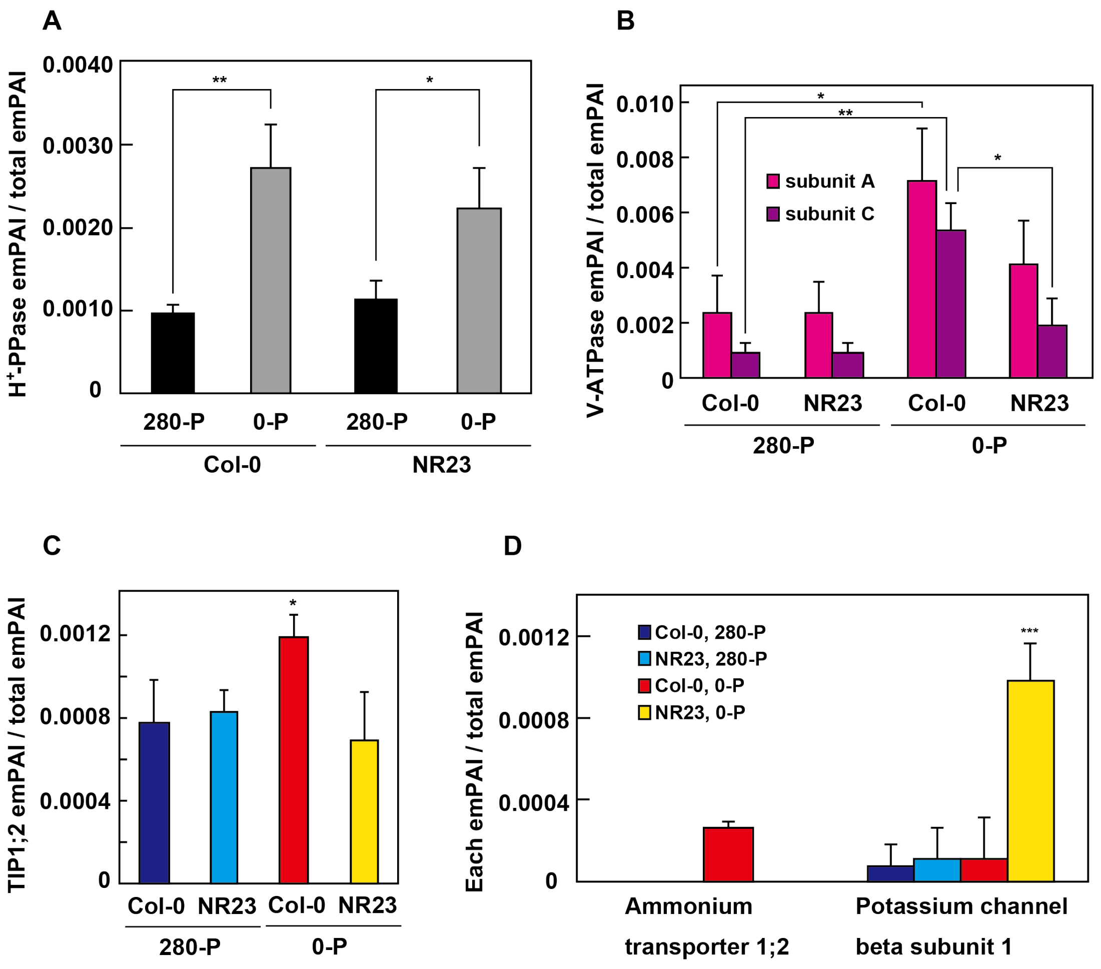

Fig. S9. Protein amounts of aquaporins, H^+-pyrophosphatase, vacuolar H^+-ATPase, ammonium transporter1;2, and potassium channel beta subunit 1 changed markedly under Pi-deficient conditions. Amounts of vacuolar membrane H^+-pyrophosphatase (H^+-PPase) (A), subunits A and C of vacuolar membrane H^+-ATPase (V-ATPase) (B), vacuolar membrane aquaporin TIP1;2 (C), ammonium transporter 1;2 (D), and potassium channel beta subunit 1 (D) in Col-0 and NR23 under normal (P, 280 μM; 280-P) and Pi-deficient (P, 0 μM; 0-P) conditions. Preparation of membrane fractions and proteomic analysis were carried out as described in the Materials and Methods. Analyses were performed independently and in triplicate. *P < 0.05, **P < 0.01, ***P < 0.005.

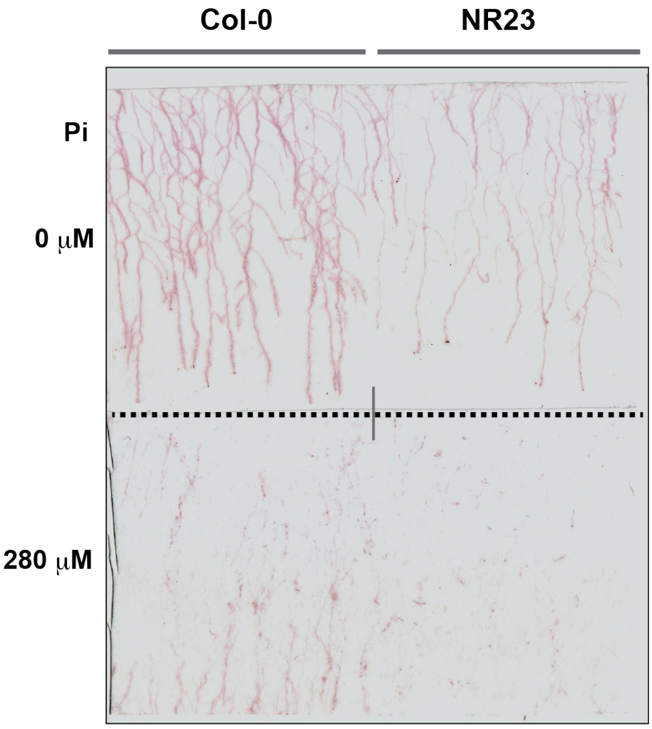

Fig. S10. Secretion of acid phosphatases was reduced in NR23. To determine the acid phosphatase activity, seedlings were grown for 20 d in agar plates with or without Pi, which were set vertically. Filter papers were soaked in reaction medium containing substrate, placed on the agar plates, and then incubated for 3 min. The stained filter papers were photographed.
